# Quantitative proteomic analysis of *Arabidopsis thaliana* with different levels of p*hospholipid:diacylglycerol acyltransferase1* expression

**DOI:** 10.1186/s12864-025-12041-7

**Published:** 2025-09-29

**Authors:** Artur Piróg, Sylwia Klińska-Bąchor, Bartosz Głąb, Sara Kędzierska, Katarzyna Jasieniecka-Gazarkiewicz, Antoni Banaś, Sachin Kote

**Affiliations:** 1 International Centre for Cancer Vaccine Science, University of Gdansk, Kladki 24, Gdańsk, 80-822 Poland; 2 Intercollegiate Faculty of Biotechnology, University of Gdańsk and Medical University of Gdańsk, Abrahama 58, Gdańsk, 80-307 Poland

**Keywords:** Lipid metabolism, Phospholipid:diacylglycerol acyltransferase, Proteomics, Stress response

## Abstract

**Background:**

Phospholipid:diacylglycerol acyltransferase1 (PDAT1) enzyme is known to play an important role in lipid metabolism. Initially, it was suggested that the construction of *PDAT1* overexpressors may result in plant varieties with increased oil content. Contrary to those expectations, *Arabidopsis thaliana* lines overexpressing *AtPDAT1* did not exhibit an increased accumulation of the triacylglycerol pool. However, the overexpressing lines exhibited accelerated growth rate, increased seed yield, and when subjected to cold treatments, they had greater thermotolerance, whereas knock-out line were more susceptible than the wild-type.

**Result:**

In this work, we have performed a comparative proteomic analysis among wild-type, *AtPDAT1-*overexpressing and knock-out lines of *Arabidopsis thaliana.* For overexpressor lines we have observed significant changes in protein profiles related to processes that may partially explain increased plant vitality. Among others, we noticed elevated levels of subsequent groups of proteins: plastoglobule structural components and associated enzymes, proteins involved in photoprotection and in autophagy. The overexpressor lines were also characterized by upregulated level of proteins involved in abiotic stress responses, whereas the level of the proteins involved in biotic stress responses was downregulated. The opposite results were detected for the knock-out line.

**Conclusion:**

These results reveal a wide range of proteomic changes that reflect cellular alterations - some of which are supported by previous physiological and biochemical studies - induced by the genetic manipulation of a single gene encoding an enzyme directly involved in lipid metabolism. The results suggest a significant role of *At*PDAT1 in plants responses to both biotic and abiotic stresses, highlighting its potential as a target for further research.

**Supplementary Information:**

The online version contains supplementary material available at 10.1186/s12864-025-12041-7.

## Introduction

Triacylglycerol (TAG) is a major energy reserve and carbon source in most plant species. These highly energy-dense compounds are accumulated mainly in seeds, while in vegetative tissue they constitute only a negligible fraction of acyl lipids. Biosynthesis of TAG takes place in the endoplasmic reticulum and might be catalyzed by two distinct enzymes. The acyl-CoA:diacylglycerol acyltransferase (DGAT) transfers an acyl group from the cytosolic pool of acyl-CoAs to the diacylglycerol (DAG), acylating the *sn* −3 position of DAG [1–3]. The other enzyme, phospholipid:diacylglycerol acyltransferase (PDAT) transfers acyl groups from phospholipids (mainly from phosphatidylethanolamine or phosphatidylcholine, PE and PC) to the *sn* −3 position of DAG [4–6].

In the early 2000 s, significant attention of the scientists was focused on creation of plants with enhanced ability for TAG production. Overexpression of *DGAT* genes in Arabidopsis brought positive results. The overexpressors accumulated higher amount of oil and produced bigger seeds [7] than wild-type. After discovering PDAT enzymes, scientists truly believed that elevation of activity of this enzyme by overexpression will bring new promising cultivars. Data from the experiment regarding overexpression of *PDAT* gene in yeast cells in which TAG accumulation increased by up to 150% compared to control lines, further reinforced these expectations [5]. Nevertheless, first study done by Ståhl et al. [6] revealed that the fatty acid and lipid composition, as well as the amounts of lipids per fresh weight in Arabidopsis plants overexpressing *AtPDAT1* did not significantly differ from those of the control plants. Only later research focusing on plant morphology showed that overexpressing *AtPDAT1* increased the growth rate of Arabidopsis transformants, including period of germination and stage of vegetative development [8]. The *AtPDAT1* overexpression transformants also had higher seed yield and oil content, after long-term subjection to cold or after cultivation under standard conditions [9].

The first assumptions that PDAT may also play an essential physiological role, especially in plant stress responses, appeared in 2017. In that year, Yuan et al. [10] showed that different *PDAT* isoforms are upregulated under distinct stress conditions. In *Camelina sativa*, expression levels of the *PDAT* genes increase by as much as 3.3, 3.5 or 5.1-times when *Camelina* plants were subjected to osmotic, cold or drought stress, respectively [10]. A similar up-regulation of *PDAT1* has been observed in *Olea europaea* cultivar Picual and in *Xanthoceras sorbifolium* subjected to low temperatures [11, 12]. Further, in vitro studies based on *pdat1* mutants (knock-out) provided knowledge about the role of PDAT in high-temperature tolerance and its indispensability for TAG production during the adverse conditions [13]. Arabidopsis *pdat1* plants were more sensitive to heat stress, were not able to accumulate TAG and were characterized by reduced number of surviving seedlings in comparison with control lines. Further study, done by Demski et al. [14] confirmed above statements by testing *AtPDAT1*-overexpressing and *pdat*1 (knock-out) lines in elevated temperature, in in vitro growth conditions. This group of scientists also evaluated the behavior of the aforementioned lines under cold stress. Cold stress conditions caused reduced growth and pigment loss in wild-type and knock-out lines, whereas *AtPDAT1*-overexpressing plants maintained their pigmentation. Notably, the leaves of these plants were four times heavier than those of wild-type plants. Augmented biomass content was not only confirmed for plants’ lines cultivated in vitro but also those grown in pots - both subjected to standard and cold stress conditions [14]. Expression analysis revealed that both low and high temperature stress conditions upregulate expression of *PDAT* genes not only in *AtPDAT1*-overexpressing lines but also in control plants [9, 14]. *AtPDAT1* overexpression also increased expression level and activity of other acyltransferases involved in lipid metabolism [9, 14]. Furthermore, as demonstrated in our previous study, increased phospholipid remodeling resulting from *AtPDAT1* overexpression enhances autophagy flux [9].

Research conducted so far clearly indicate that overexpression of *AtPDAT1* in Arabidopsis boosts lipid metabolism and can induce efficient abiotic stress response. In general, *AtPDAT1*-overexpressing lines can reduce their temperature sensitivity (when subjected to cold), augment biomass production and increase seed yields and oil production. Nevertheless, based on the results collected so far, the observed effect cannot be directly attributed to or fully explained by the currently known PDAT activity. It is likely that its upregulated expression indirectly influences various processes, such as growth stimulation, membrane lipid metabolism, autophagy and others. In this manuscript, for the first time, we investigate the changes at the protein level triggered by genetic manipulations of *AtPDAT1* overexpression and knock-out in Arabidopsis plants grown under standard conditions to explain observed phenotypes and to indicate how the introduced mutations affect the accumulation of other proteins.

## Experimental procedures

### Plant material and growth conditions

*Arabidopsis thaliana* ecotype Columbia-0 (Col-0) plants were used as control/wild-type plants. The transformation method of Arabidopsis wild-type plants with *AtPDAT1* gene (*accession number: At5g13640)* was previously described by Ståhl et al. [6] and Banaś et al. [8]. Two tested *PDAT*-overexpressing lines, designed during the above-mentioned study were kindly provided by both scientists’ teams. In both tested, transformed lines, overexpressing the *AtPDAT1* gene was constructed in the pART27 vector, and expressed under the 35 S Cauliflower Mosaic Virus promoter (CaMV). The used knock-out mutant of *pdat1* (SALK_032261) was obtained from the Arabidopsis Biological Resource Center (The Ohio State University, Columbus, Ohio, USA). The homogeneity of the knock-out line was previously confirmed by polymerase chain reaction screening [14].

Seeds of *A. thaliana* (control) line, two *AtPDAT1*-overexpressing lines (OE1 and OE2) and one knock-out line (KO) were planted into the pots with soil. Before sowing, they were stratified at 4 °C for 48 h. In conducted experiments, plants were cultivated under standard conditions in growth chamber at a constant temperature of 22 °C ± 1 °C with 60% relative humidity and a photoperiod of 16 h of light (120 µmol photons m^−2^ s^−1^)/8 h of darkness. Materials for further protein extraction (100 mg of Arabidopsis leaves tissue) were collected about 4.5 weeks after sowing, when the first flower emerged. Collected leaves tissue fragments were frozen in liquid nitrogen and stored at −80 °C for further analysis.

### Sample preparation for proteomic analysis

For all the analyzed conditions, five biological replicates (leaf tissue from five individual plants) were used. Analyses of *AtPDAT1* overexpression and knock-out were performed separately – wild type samples were prepared independently for each experiment. For each sample, 100 mg of Arabidopsis rosette leaf was placed in 2 ml plastic tube with screw, together with 250 mg of glass beads (0.10–0.11 mm; Sartorius Stedim) and frozen in liquid N_2_. Samples were homogenized by shaking in Mini-BeadBeater (BioSpec) 10 × 20 s, refreezing the tubes in liquid N_2_ in between to avoid thawing. Powdered sample was precipitated by the addition of 1500 µl ice-cold solution made of acetone with 10% trichloroacetic acid and 0.07% β-mercaptoethanol (β-Me), and overnight incubation in −20 °C. Subsequently, samples were spun down for 10 min at 5000 x g at 4 °C. The supernatant was removed. Precipitate was washed twice with acetone + 0.07% β-Me and dried under nitrogen stream. The precipitate, together with glass beads, was resuspended in 100–200 µl (depending on pellet size) of extraction buffer, composed of 50 mM Tris-HCl pH 8.0 (MP Biomedical, Santa Ana, California, USA), 8 M urea, 1 mM dithiothreitol, + 1x protease inhibitor cocktail EDTA-free (Roche). Then, samples were vortexed and sonicated for 2 h. After that, glass beads and precipitate were spun down, and 75 µl of supernatant was transferred to fresh tube. Another 75 µl of extraction buffer was used to wash the beads by vortexing and sonication for 5 min. Both portions of buffer were combined. Protein concentration was measured using protein BCA assay [15]. The resulting samples exhibited a protein concentration in the range 2–3 µg/µl.

For protein digestion, 60 µg of protein was collected from each sample and diluted to 80 µl with 8 M Urea/100 mM NH_4_HCO_3_. Subsequently, proteins were reduced by addition of dithiothreitol to 20 mM concentration and incubation for 30 min at 37 °C, and alkylated by addition of iodoacetamide to 60 mM concentration and incubation for 30 min at 37 °C in the dark. Then, urea was diluted to 1 M with 100 mM NH_4_HCO_3_ and 2 µg of trypsin (Promega) was added. Digestion was carried out overnight at 37 °C. Peptides were desalted by C18 MacroSpin columns (Harvard Apparatus) and dried under vacuum. Before analysis, dried peptides were resuspended in loading buffer (2.5% acetonitrile in 0.08% trifluoroacetic acid (TFA). Peptide concentration was measured by absorbance at 280 nm. Peptides were diluted with loading buffer to obtain concentration of 300 ng/µl. Before analysis, 0,5 µl of iRT peptide solution (Biognosys), prepared according to manufacturer’s instructions were added to each sample.

### Peptide fractionation

For peptide fractionation, digested peptides were resuspended in 2.5% acetonitrile and 0.08% trifluoroacetic acid and applied on two High pH Reversed-Phase Peptide Fractionation Kit (Pierce) columns, using 90 µg peptides per column. Fractionation was performed according to the manufacturer’s instructions resulting in 8 main fractions, flow-through and wash fractions. The respective fractions were combined and dried under vacuum. Before analysis, dried peptides were resuspended in loading buffer (2.5% acetonitrile in 0.08% TFA). These samples were used solely for qualitative data-dependent analysis (DDA) runs aimed at collecting an experimental spectra library for subsequent DIA data analysis. Before analysis, 0.5 µl of iRT peptide solution (Biognosys), prepared according to manufacturer’s instructions were added to each sample.

### Liquid chromatography/mass spectrometry analysis

Liquid chromatography conditions were identical for data-dependent acquisition (DDA) and data-independent acquisition (DIA) experiments. Samples were separated on Thermo Scientific RSLC 3000nano LC system, coupled to a Thermo Scientific Orbitrap Exploris 480 mass spectrometer. Chromatography was performed by concentrating peptides on 0.3 × 5 mm C18 PepMap trap column (Thermo Scientific) at a flow rate of 5 µl/min for 10 min and further separating them on 0.075 mm x 250 mm, 2 μm particle size C18 PepMap RSLC column (Thermo Scientific). Loading buffer was identical to sample dissolution buffer, mobile phase A was 2.5% acetonitrile and 0,1% formic acid in water, and mobile phase B was 80% acetonitrile in 0.1% formic acid in water. Peptides were separated by increasing mobile phase B from 2.5 to 40% in 90 min followed with washing with 99% B and re-equilibration to initial conditions.

DDA data were acquired using a full scan with 60,000 resolution, a 350–1200 Th mass range, 300% AGC target and an automatic maximum injection time. Full scans were saved as profile spectra. Ions with charge state from 2 to 6 and a minimum intensity of 5000 were selected for fragmentation. Dynamic exclusion was set to 20 s within +−10 ppm range from fragmented precursor and its isotopes. Isolation window was set to 2 Th, resolution to 30,000 and normalized HCD fragmentation energy to 30%. AGC target was set to “Standard” and maximum ion injection time to Auto. Scan range was set to “First mass” mode with starting m/z of 110 Th.

DIA data were acquired using a full scan with 60000 resolution, a 350–1450 Th mass range, 300% AGC target and a 100 ms maximum injection time. DIA method used 62 × 12 Th windows with 1 Th overlaps, covering mass range from 350 to 1100 Th. Resolution was set to 30000, AGC to 1000% and HCD fragmentation energy to 30%, using 2 + as the default ion charge state. Spectra were saved in profile mode.

### Peptide identification

Raw data files (DDA and DIA runs) were converted to.mzML files using MSConvert with centroiding. Converted MS data were searched using MSFragger 3.4 embedded in Fragpipe suite (v. 17.1) against complete *Arabidopsis thaliana* Uniprot database (SwissProt + Trembl, all isoforms) concatenated with indexed retention time (iRT) peptide sequences, decoy reverse sequences and contaminants. The search database was constructed in Fragpipe. Additional DDA-like data was extracted from DIA (data independent acquisition) files by DIA-Umpire signal extraction engine integrated in Fragpipe. The following search settings were used for MSFragger: precursor mass tolerance was set to +/−10 ppm and fragment mass tolerance was automatically optimized. Enzyme digestion was set to trypsin and peptide length was set from 7 to 40 amino acids. Peptide mass range was set from 500 to 5000 Dalton. Variable modifications were set to: methionine oxidation, protein N-term acetylation and peptide N-term propionaldehyde (artifact of acetone treatment). The maximum allowed fragment charge was set to 2. Data were subjected to mass recalibration, and automatic parameter optimization setting was used to tune fragment mass tolerance. Search result filtration and validation were done automatically by Philosopher using Percolator and MSBooster. EasyPQP was used to create the spectral library for further use in DIA-based quantification.

### Protein quantification

DIA data processing was performed using DIANN v. 1.8. Empirical spectral library was created from search results of analyzed samples, peptide fractions and search results of previously performed preliminary experiments. This library was supplemented with in silico predictions performed by DIANN using tryptic peptides with a maximum of one missed cleavage, length range from 7 to 30 amino acids and methionine oxidation allowed as a variable modification.

For final quantification, all.mzML files were converted to.dia files. MBR option was selected. Precursor FDR was set to 1%. Robust LC (high accuracy) option was selected. MS2 and MS1 mass accuracies, and scan window were set to 13 ppm, 13 ppm and 7, respectively. Values were chosen as suggested by DIANN after analyzing each run separately. Retention time dependent cross-run normalization was applied to data. Other settings were left as default.

Standard protein quantification was performed using the diann-r R library and the MaxLFQ algorithm. To quantify proteins exhibiting extreme fold changes, which may not be properly quantified by MaxLFQ, we have additionally performed quantification based on simple peptide intensity summing. Summing-based quantifications were performed using custom Python script.

First experiment (analysis of the effect of overexpression) involved 3 conditions – wild-type and two overexpressor lines.

For MaxLFQ protein quantification, significantly regulated proteins were defined as fulfilling all of the following criteria:

1. Valid at 1% FDR in any of two-sample Welch test with permutation-based FDR correction.
2. Valid at 1% FDR in ANOVA (all three conditions) with permutation-based FDR correction.
3. Exhibited twofold or more difference in at least one inter-group comparison.
4. Had a minimum 4 valid values per experimental condition.

For peptide intensity summing quantification, validation was done by manual inspection of peptide intensity profiles and statistical tests were used to identify candidates.

Second experiment (analysis of the effect of knock-out) involved 2 conditions – wild-type and one knock-out line.

For MaxLFQ protein quantification, significantly regulated proteins were defined as fulfilling all of the following criteria:

1. Valid at 1 % FDR in two-sample Welch test with permutation-based FDR correction.
2. Minimum 0.8 (log2) abundance change between groups.

For peptide intensity summing quantification, analysis was done similarly as in first experiment.

Peptide profiles of selected proteins are included in supplementary data.

### Gene ontology analysis

Before annotation, Uniprot protein identifiers were translated to TAIR identifiers (www.arabidopsis.org) as they are most used in Gene Ontology for *Arabidopsis thaliana.* Complete ontology was downloaded from geneontology.org. Term annotation and visualization of quantitative changes in the context of ontology terms were performed using custom Python scripts. For generation of the global ontology term quantitative heatmaps, protein groups belonging to single term were summarized by simple average (no weighting) or weighted average using -log10(ANOVA p-value) as weight (significance weighting).

### Proline content analysis

To determine the proline content in the tested plant lines about 100 mg of fresh leaves were collected and frozen in liquid nitrogen. Subsequently, the plant material was homogenized in 80% ethanol using glass beads and sand. Supernatants derived after homogenization in BeadBeater (3 min) followed by sonification (20 min) and centrifugation (15 min, 10.000 x g) were directly used for proline content analysis. Further steps were conducted according to methods described by López-Hidalgo et al. [16]. The results are presented in Supplementary Fig. S6.

## Results

### Data quality

We successfully conducted a comparative analysis of proteomes of wild-type, *AtPDAT1* overexpressor, and knock-out plants. The sample preparation method described here ensures a high degree of homogenization before performing protein extraction, which is a prerequisite for plant tissues. In our initial efforts, suboptimal homogenization led to irreproducibility, probably due to the variable level of extraction from more fibrous components of the leaves. Prolonged acetone precipitation and extraction were necessary to remove substances that would otherwise interfere with LC/MS analysis of extracted peptides, as substantial amount of plant metabolites separate efficiently on reverse-phase columns widely used in proteomic analyses. Figure S1 shows the overall quality metrics of obtained experimental data. Overall, we quantified 6023 protein groups, 5196 of which were without missing value introduction. Such in-depth analysis provided an opportunity to obtain data on less-abundant proteins. We consider this a remarkable success, since plant proteome sample preparation is generally complex and prone to irreproducibility [17].

### Protein quantification results

**Fig. 1:**
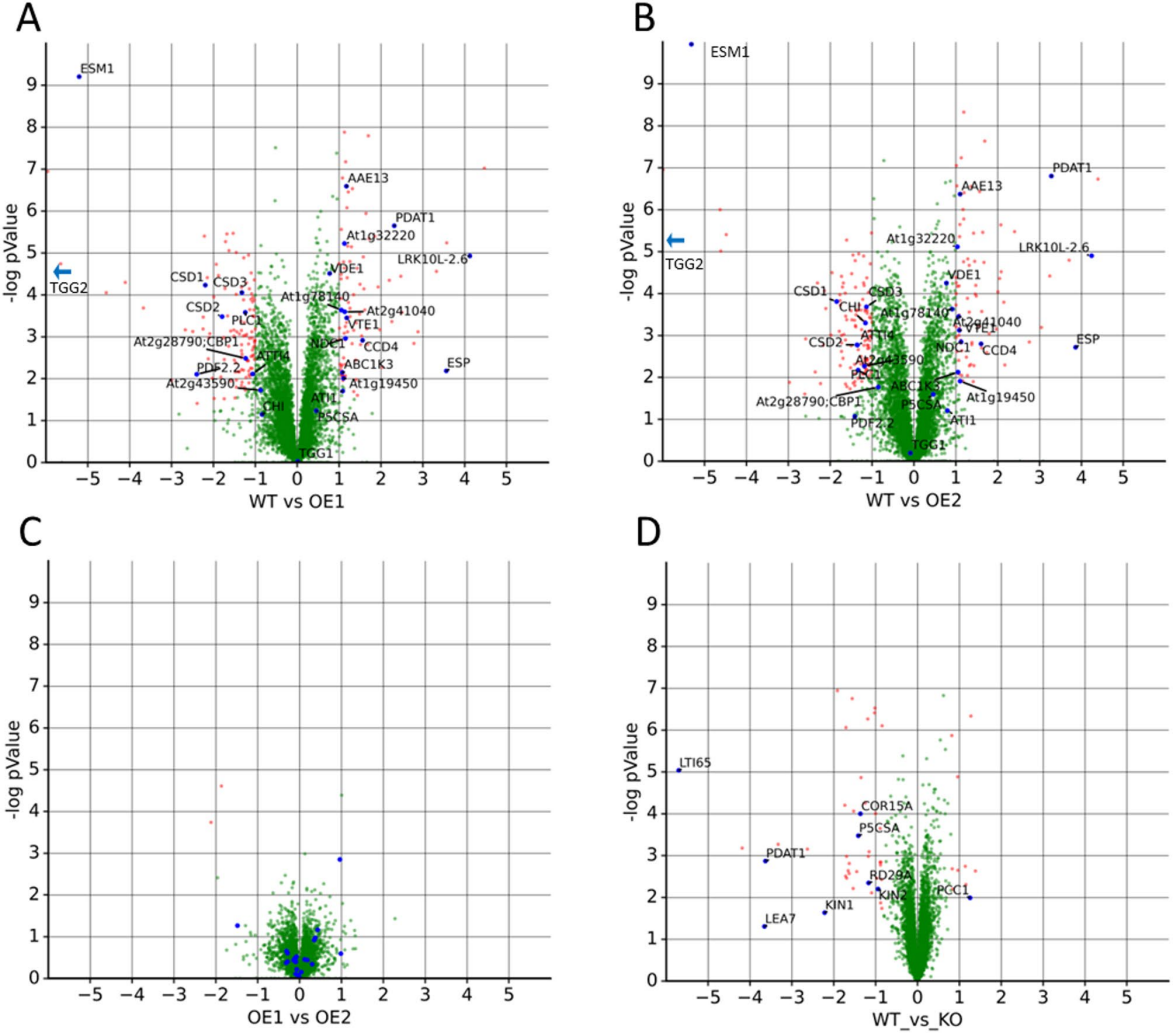
Overview of obtained results - volcano plots showing differences in proteomes of analyzed plants. Red dots denote significantly altered proteins. Proteins referred in text are marked by blue dots. **A** Wild-type vs. overexpressor (1) **B**) Wild-type vs. overexpressor (2) **C**) Overexpressor 1 vs. overexpressor 2. **D** Wild-type vs. knock-out. For visualization purposes, data were imputed assuming left-censored missing value distribution for proteins found to be practically absent in some conditions. Quantified protein groups are annotated with corresponding gene names for clarity

Two individual lines of *AtPDAT1* overexpressor were found to be almost identical (Fig. 1C), which ensured that the observed effects were indeed the result of *AtPDAT1* overexpression and were not the effects of off-target modifications of genome. We have observed considerable changes in proteome (Fig. 1AB), resulting in detection of 247 significantly regulated protein groups after analysis and filtering of the results. The effects observed in the *pdat1*-knockout line were overall of lesser magnitude, resulting in 118 significantly regulated protein groups, even after less stringent filtering, and much fewer proteins showed extreme abundance changes (Fig. 1D). All significantly regulated proteins are summarized in Supplementary Table S1 (overexpression) and Table S2 (knockout).

**Fig. 2:**
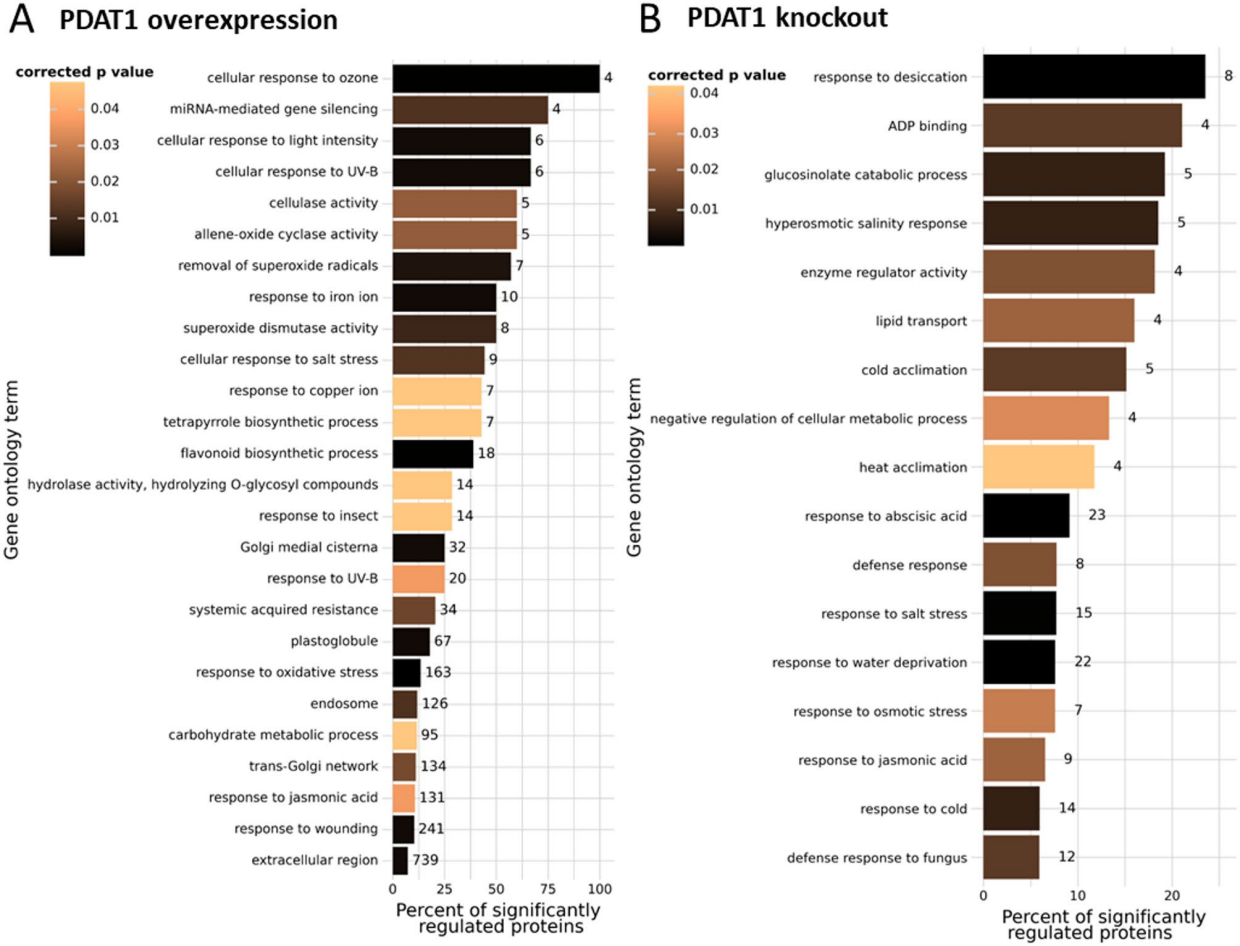
Overview of Gene Ontology terms enriched in set of significantly regulated proteins. **A** In analysis of *AtPDAT1* overexpressors. **B** In analysis of the *pdat1* knock-out line. Bar width denotes the percentage of significantly regulated proteins among all proteins detected in particular term. Color mapping corresponds to Benjamini-Hochberg corrected p-value of Fisher test for enrichment versus all detected proteins as background. Numeric labels show total number of proteins detected for particular term

The majority of the changes observed in *AtPDAT1-*overexpressing lines were not directly related to lipid metabolism. This pattern may indicate that *AtPDAT1* overexpression affects a broader range of physiological processes in Arabidopsis. To obtain an overall view of the affected processes, we performed a gene ontology (GO) analysis of the results. Figure 2 presents the Biological Process (BP), Molecular Function (MF) and Cellular Component (CC) terms, that were enriched in a set of significantly regulated proteins. The effect of *AtPDAT1* overexpression was clearly much stronger than the knock-out mutation. This can be seen in Fig. 1, as magnitudes of protein expression differences between WT and overexpressors (panels A, B) are much higher than when comparing WT plants to knock-outs (panel D). In the knock-out and overexpressor lines, processes and activities related to stress response were clearly affected. In *AtPDAT1* overexpressors, however, a much wider variety of additional functional and structural terms was observed, as shown in Fig. 2, including activities related to plant cell wall biogenesis and maintenance and plastoglobule contents.

**Fig. 3:**
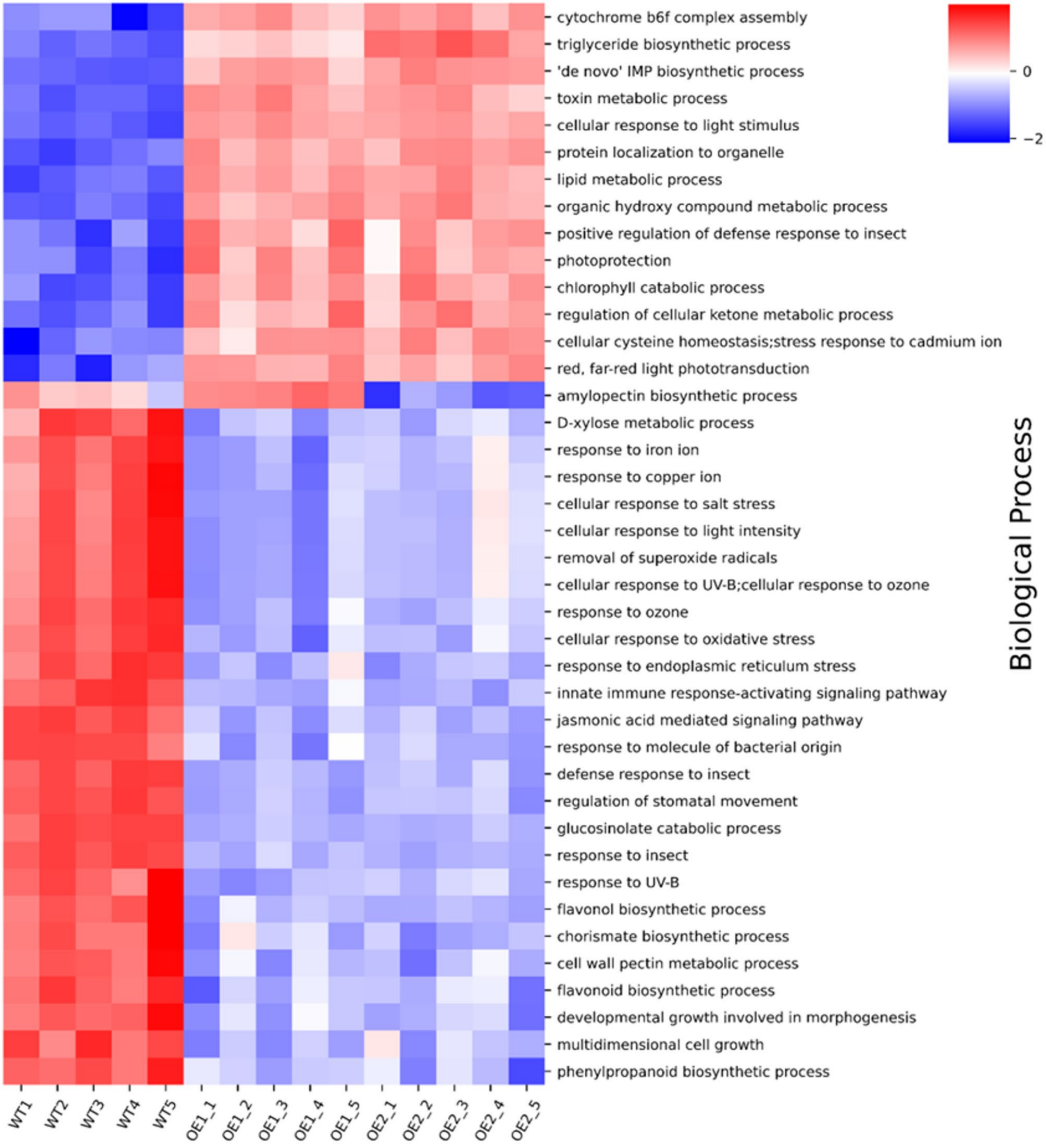
Heatmap overview of changes in Biological Process Gene Ontology terms between wild-type (WT) and ***AtPDAT1***-overexpressing lines (OE1, OE2). Term regulation was calculated as a weighted average per sample, followed by row-wise Z-score normalization. Red color denotes overall upregulation of term, and blue color overall downregulation of term

Enrichment analysis alone, while useful, may lead to considerable oversimplification, due to loss of information about protein groups which exhibited a sub-significant change, but when averaged together as one process or activity, they can provide valuable information. Additionally, simple averaging may be substituted with weighted average with statistical significance component (in this case -log10(ANOVA p-value)). This approach leads to two different kinds of answers: without weighting, one can visualize groups showing significant global change, whereas using weights highlights terms that do not change overall but contain a significant number of regulated proteins. We have used this information as a guideline for describing the observed effects and deciphering the possible underlying mechanism. As before, this gave clear results for *AtPDAT1*-overexpressing plants. Visualization for BP gene ontology terms with significance weighting (see Methods for details) are shown in Fig. 3. Complete results, generated with and without significance weighting are shown in Supplementary Fig. S2. In the case of data from the *pdat1* mutant, there were very few visibly affected terms, and resulting changes were mostly caused by a single protein or several protein isoforms. Results for unweighted term averages are shown in Supplementary Fig. S3. Obtained results confirm and extend observations from enrichment analysis – most affected processes were related to stress response or regulation of plant growth and development. Selected terms are visualized on the individual protein level in Supplementary Fig. S4. For both experiments, proteins mentioned and examined in detail, further in the discussion section, are summarized in Table 1, and peptide-level quantification plots are presented in Supplementary Fig. S5.

**Table 1:** Summary of quantification of protein groups mentioned in discussion. **A** comparison of wild-type and overexpressor lines, **B** comparison of wild-type and knock-out line. Difference is presented as log2 fold change and q-value denotes p-value corrected for multiple testing by permutation, as described in Methods. Green color indicates validity (q-value <0.01, difference >1, log_2_ scaled). For several proteins only approximate values are presented (q-value is n/a), as respective proteins were undoubtedly detected with multiple peptides in one of the conditions and were almost undetectable in other. Therefore, exact fold change could not be calculated

***A - Comparison of wild-type and overexpressor lines***						
**q-value OE1/WT**	**Difference OE1 - WT**	**q-value OE1/OE2**	**Difference OE1 - OE2**	**Protein name**	**Gene**	**TAIR Gene symbol**
n/a	>-5	n/a	~0	Myrosinase 2	TGG2	AT5G25980
0.00	-5.21	1	0.11	GDSL esterase/lipase ESM1	ESM1	AT3G14210
0.00	-2.19	1	-0.35	Superoxide dismutase [Cu-Zn] 1	CSD1	AT1G08830
1.56E-03	-2.40	1	-0.99	Defensin-like protein 2	PDF2.2	AT2G02100
2.00E-04	-1.79	1	-0.43	Superoxide dismutase [Cu-Zn] 2, chloroplastic	CSD2	AT2G28190
3.93E-03	-1.06	1	0.29	Defensin-like protein 196	ATTI4	AT2G43535
2.79E-04	-1.24	1	0.09	Phosphoinositide phospholipase C1	PLC1	AT5G58670
1.02E-02	-0.87	1	0.31	Endochitinase At2g43590	At2g43590	AT2G43590
3.72E-02	-0.84	1	0.32	Endochitinase CHI	CHI	AT2G43570
2.26E-04	-1.32	1	-0.19	Superoxide dismutase [Cu-Zn] 3	CSD3	AT5G18100
1.59E-03	-1.23	1	-0.38	Pathogenesis-related thaumatin superfamily protein	At2g28790	AT2G28790
5.92E-02	0.46	1	-0.005	Delta-1-pyrroline-5-carboxylate synthase A	P5CSA	AT2G39800
9.57E-01	0.01	1	0.10	Myrosinase 1	TGG1	AT5G26000
2.50E-04	0.78	1	0.00	Violaxanthin de-epoxidase, chloroplastic	VDE1	AT1G08550
8.08E-03	1.09	1	0.29	ATG8-interacting protein 1	ATI1	AT2G45980
2.24E-04	1.06	1	0.14	Uncharacterized methyltransferase At1g78140 chloroplastic	At1g78140	AT1G78140
0.00	1.13	1	0.09	Uncharacterized protein At1g32220, chloroplastic	At1g32220	AT1G32220
3.60E-03	1.08	1	0.03	Protein ACTIVITY OF BC1 COMPLEX KINASE 3 chloroplastic	ABC1K3	AT1G79600
2.47E-04	1.14	1	0.07	Uncharacterized methyltransferase At2g41040 chloroplastic	At2g41040	AT2G41040
2.33E-04	1.18	1	0.10	Tocopherol cyclase, chloroplastic	VTE1	AT4G32770
0.00E+00	1.18	1	0.07	Malonate--CoA ligase	AAE13	AT3G16170
4.61E-03	1.11	1	0.00	Sugar transporter ERD6-like 4	At1g19450	AT1G19450
8.62E-04	1.15	1	0.03	Alternative NAD(P)H-ubiquinone oxidoreductase C1 chloroplastic/mitochondrial	NDC1	AT5G08740
5.63E-04	1.56	1	-0.04	Probable carotenoid cleavage dioxygenase 4 chloroplastic	CCD4	AT4G19170
0.00	2.32	0.11	-0.96	Phospholipid:diacylglycerol acyltransferase 1	PDAT1	AT5G13640
0.00	4.12	1	-0.12	Protein SUPPRESSOR OF NPR1-1 CONSTITUTIVE 4	LRK10L-2.6	AT1G66980
n/a	>5	n/a	~0	Epithiospecifier protein	ESP	AT1G54040
***B -Comparison of wild-type and knock-out line***						
**q-value KO/WT**		**Difference KO - WT**		**Protein name**	**Gene**	
n/a		<-5		Low-temperature-induced 65 kDa protein	LTI65	AT5G52300
0.002		-1.17		Low-temperature-induced 78 kDa protein	RD29A	AT5G52310
n/a		<-5		Late embryogenesis abundant protein 7	LEA7	AT1G52690
n/a		<-5		Stress-induced protein KIN1	KIN1	AT5G15960
2E-4		-1.36		Protein COLD-REGULATED 15A, chloroplastic	COR15A	AT2G42450
0.005		1.25617		Cysteine-rich and transmembrane domain-containing protein PCC1	PCC1	AT3G22231
0.005		-1.17252		Stress-induced protein KIN2	KIN2	AT5G15970
1E-4		-1.41269		Delta-1-pyrroline-5-carboxylate synthase A	P5CSA	AT2G39800
n/a		<-5		Phospholipid:diacylglycerol acyltransferase 1	PDAT1	AT5G13640

## Discussion

In the presented study we elucidated the potential mechanism underlying the increase of overall plant fitness by *AtPDAT1* overexpression. Although PDAT enzymes are recognized as enzymes participating in TAG biosynthesis, the latest scientific evidence points to their extremely significant roles in vitality maintenance, yield, biomass production and stress adaptation. Our results clearly showed that overexpression of *AtPDAT1* significantly affected the whole plant proteome. *AtPDAT1* overexpression affects the production of plastoglobules, and the enzymes associated with this process. It positively affected the levels of proteins participating in stress response, involved in photoprotection and autophagy (Table 1.). Knock-out of this gene had a milder effect on the protein profile of Arabidopsis than its overexpression. This modification resulted in a proteome comparable to that of wild-type plants; however, the knock-out line exhibited some changes that may affect their tolerance to abiotic and biotic stress, which contrasted with those observed in the overexpressor lines (Table 1.).

As the leaves are not specialized in TAG accumulation, the importance of PDAT enzyme in photosynthetic tissue is assumed to be related to the remodeling of membrane lipids (phospholipids) which are crucial to maintaining membrane homeostasis, proper structure and fluidity, as well as to the utilization of oxygenated fatty acids and sequestration of toxic lipid intermediates [6, 18, 19]. Presumably, the TAG pool formed in leaves is efficiently degraded via TAG-lipases – such as SDP1 (Triacylglycerol lipase SDP1) and then converted into sugars in the process of gluconeogenesis from malate which is formed in the glyoxalase cycle from acetyl-CoA produced during β-oxidation of fatty acids released from TAG pool [20, 21]. The direct correlation between overexpression of *AtPDAT1* gene and elevated expression of *SDP1* has already been shown, as well as its further connection with PXA1 (ABC transporter D family member 1) responsible for fatty acids transfer to peroxisomes [9, 22]. Based on our proteomic results, we noticed the significantly elevated level of Sugar transporter ERD6-like 4 protein in overexpressor lines, which might be one of the pieces of evidence supporting this hypothesis of conversion of fatty acids into sugars, which are then transferred to various organs and used as a building material (Table 1.). So far this speculation might be also one of the explanations for augmented and accelerated growth of overexpressor lines, which at the early stages of development were characterized by approximately 30–50% higher biomass content than control plants [9, 14].

The evidence for boosted lipid metabolism, besides the detection of an increased amount of protein with PDAT activity (Table 1.), is the increased fatty acid biosynthesis which is utilized during lipid remodeling. In overexpressor lines, the elevated amount of Malonate-CoA ligase has been identified. This enzyme can produce malonyl-CoA, a compound used as a precursor for the *de novo* synthesis and elongation of fatty acids [23] or used for the formation of secondary metabolites such as flavonoids, isoflavonids, flavonoid glycosides [24]. Due to the observed reduced amount of the protein involved in flavonoid and flavon biosynthesis (Supplementary Figure S2; S4), the majority of the generated malonyl-CoA is likely utilized exclusively in fatty acid biosynthesis.

In the tested overexpressor lines, we detected an increased level of several protein components of plastoglobules (Table 1.; Supplementary Fig. S2). Plastoglobules are globular compartments enclosed by a galactolipid layer and containing neutral lipids, including triacylglycerols and carotenoids, together with enzymatically active proteins [25].

As previously described, these structures may play an essential role in plant response to abiotic stress, as their quantity rises during adverse growth conditions: salt stress, drought or presence of heavy metals [26, 27]. Our proteomic analysis shows an increased amount of proteins associated with plastoglobules: NDC1 - Alternative NAD(P)H-ubiquinone oxidoreductase C1, chloroplastic/mitochondrial, CCD4 - Probable carotenoid cleavage dioxygenase 4, chloroplastic, ABC1K3 - Protein ACTIVITY OF BC1 COMPLEX KINASE 3, chloroplastic, VTE1 - Tocopherol cyclase, chloroplastic, two proteins with methyltransferase type 11 domain (products of *At1g78140* and *At2g41040* genes) and protein with NAD-dependent epimerase/dehydratase domain (*At1g32220* gene product); [28]. NDC1 is involved in the regeneration of the reduced form of plastoquinone and participate in phylloquinone production [29]. VTE1 is an enzyme which catalyzes the second to last step of α-tocopherol biosynthesis [30], whereas CCD4 participate in carotenoids metabolism [31]. Plastoquinone (PQ) can locate in various lipid structures such as thylakoid membrane, plastoglobules and the chloroplast envelope. Mainly it locates in thylakoid membrane, participating in electron transport and photosynthesis. However, PQ is also localized in plastoglobules, especially under stress conditions, where it may function as a non-photoactive PQ reservoir that supports photosynthetic efficiency under stress conditions and when photoactive PQ is damaged. PQ also plays a significant role in non-photochemical quenching, has strong antioxidant activity that scavenger reactive oxygen species and mitigates lipid peroxidation, contributing to the photoprotection mediated by β-carotene in PSII and tocopherols in the thylakoid membrane [32]. Boosted photoprotection mechanism in *AtPDAT1*-overexpressing lines is also supported via elevated amount of Violaxanthin de-epoxidase, chloroplastic (VDE) which is protein protecting from photodamage by dissipating excessive amounts of light energy as heat and converting violaxanthin to zeaxanthin under high light stress [33]. Enhanced photoprotective mechanisms and/or photosynthetic efficacy may contribute to the observed higher seed productivity and plant biomass of *PDAT1*-overexpressing lines [8, 9, 14].

As previously observed, *AtPDAT1*-overexpressing lines are characterized by elevated level of ATG8 protein associated with autophagy process [9, 14]. Autophagy is a cellular process involved in the degradation and recycling of damaged, unnecessary proteins or cellular components. ATG8-PE complex, involved in this process, acts as a docking platform for cargo intended for degradation [34]. The elevated protein level and expression of ATG8 and decreased level one of the cargo receptors – NBR1 have been already described for leaves of *AtPDAT1* overexpressor lines [9, 14]. The current proteomic analysis also revealed higher level of other cargo receptor – ATI1 (ATG8-interacting protein 1); (Table 1.). This protein is directly involved in degradation process of plastids and endoplasmic reticulum via autophagy process [35, 36]. Similarly, ATI1 like NBR1 interacts with ATG8-PE conjugates [37, 38]. Autophagy is a process directly dependent on lipids. Fan et al. [39] suggested that disruption of basal autophagy downregulate lipid turnover, therefore such disruption might also limit the utilization of acyl groups from membrane lipids for TAG biosynthesis. In the case of *AtPDAT1* overexpression it seems that such modifications simultaneously boost lipid turnover and autophagy process which drive each other, and stimulate other processes associated with lipid metabolisms [9, 14, 39].

The mechanisms described above are probably responsible for ensuring greater fitness and protection against abiotic stress, if any occur. So far, improved development of *AtPDAT1*-overexpressing lines has been noticed under standard, cold and in vitro cultivation conditions [9, 14]. Most of the detected and upregulated proteins and processes described here are strongly suggested to be major players in the adaptation and improvement of plant vitality in the presence of abiotic stresses. In contrast processes involved in biotic stress response are downregulated. Principally, the level of Phosphoinositide phospholipase C1, which is known as an important protein in plant signaling process during stress [40], declined notably in *AtPDAT1*-overexpressing lines. Among other identified proteins that differ significantly, we noticed the increased level Protein SUPPRESSOR OF NPR1-1 CONSTITUTIVE 4 (SNC1). SNC1 is a negative regulator of NPR1 (Regulatory protein NPR1) which is a key protein in systemic acquired resistance, acting by regulating *PR* (Plant Resistant) genes expression, activating defense responses [41]. In the consequence of increased SNC1, the level of proteins belonging to the PR proteins, Pathogenesis-related thaumatin superfamily protein, Endochitinases and Defensin-like protein 2 and Defensin-like protein 196, were also reduced (Table 1.). This suggests that the mechanisms and proteins involved in the defense response to pathogen is weakened in the *AtPDAT1*-overexpressing lines. This is particularly interesting in the context of further research, as the role of PDAT1 enzyme in response to this type of stress has not been yet investigated.

While *AtPDAT1* overexpressor lines showed significant alterations in proteins directly or indirectly associated with lipid metabolisms, this modification also affected levels of proteins which are not correlated with lipids or PDAT activity. Examples of such protein groups are enzymes involved biosynthesis, modification and degradation of plant cell wall polysaccharides (Supplementary Figure S2; S4). The altered proportion of the amounts of cellulose, pectin methylesterification and xylose and their reduced content can affect the elasticity and hydration of cell wall and as a result plant morphology and growth ability [42–44]. This could be manifested by the smaller rosette size in *AtPDAT1*-overexpressing lines compared to the control, starting from the onset of generative development [9, 14].

Interestingly, *AtPDAT1-*overexpressing lines also had reduced level of proteins with superoxide dismutase [Cu/Zn] (SOD) activity (Table 1.). Cu/Zn SOD is one of the many important scavenging enzymes of antioxidative defense system against reactive oxygen species, responsible for conversion of the superoxide anion to H_2_O_2_ [45]. Downregulation of these mechanisms may be caused by sufficient efficiency of the other processes leading to the minimization of oxidative stress occurrence, such as mentioned above increase of photoprotection, autophagy or elevated PDAT activity which utilize oxygenated acyl groups from phospholipids.

Additionally, a shift in the glucosinolate metabolic pathway is observed in *AtPDAT1-*overexpressing plants. Expression of the Epithiospecifier protein (ESP) is heavily upregulated and concomitantly, expression of GDSL esterase/lipase ESM1 is downregulated (Table 1.). ESP mediates formation of nitriles and epithionitriles [46], while ESM1 promotes production of isothiocyanates, represses production of nitriles and favor isothiocyanate from glucosinolate [47]. Interestingly, one of the isoforms of Myrosinase 2, enzyme which participates in glucosinolates degradation, encoded by TGG2 gene, is heavily downregulated in *AtPDAT1* overexpressor lines, while TGG1 gene product (Myrosinase 1) seem to be unaffected. A shift to less toxic nitriles may decrease the burden on plant itself, due to the lower self-toxicity of nitriles compared to isothiocyanates [48]. Moreover, as nitriles are also less toxic for mammals, it may be beneficial to investigate whether the effect of *AtPDAT1* overexpression would be similar in other *Brassicaceae* family plants of agricultural importance. However, for the plants themselves, changes in the profile of glucosinolate hydrolysis products, which are active compounds against herbivores and pathogens, may influence their response to various biotic stresses, potentially affecting their performance under field conditions.

In the case of further examining the knock-out line, we did not detect as many changes in proteins level as in the case of the overexpression lines. The overall proteome alteration observed in the knock-out line was less pronounced than that observed in the overexpression lines. Most of the detected proteins were at a level comparable with that detected for the wild-type line. In addition to the absence of *AtPDAT1*, confirming effective knock-out of the gene encoding this enzyme, we detected a decreased level of several proteins related to plant adaptation, particularly associated with low temperature stress (Table 1.). Among them, we can distinguish: Low-temperature-induced 65 kDa protein and 78 kDa protein (LTI65; LTI78), Late embryogenesis abundant protein 7 (LEA7), Stress-induced protein KIN1 and KIN2, Protein COLD-REGULATED 15 A, chloroplastic (COR15). These proteins are involved in the regulation of cold tolerance, signal transduction, membrane stabilization and enzyme protection [49–52]. This result correlates with the previously observed dwarf phenotype of *pdat1* Lines cultivated under in vitro conditions at 4 °C [14]. On the other hand, the *pdat1* knock-out showed a significantly increased level of Cysteine-rich and transmembrane domain-containing protein PCC1 (Pathogen and Circadian Controlled 1) which is recognized as a regulator of defense responses to different pathogens [53]; (Table 1.), indicating that downregulated level of *AtPDAT1* may enhance biotic stress response, in contrast to *AtPDAT1*-overexpressing lines. This further supports the above-described suggestions regarding the importance of the PDAT enzyme in the response to pathogens and herbivores. Remarkable difference between overexpressor and knock-out lines was also noticed in the level of Delta-1-pyrroline-5-carboxylate synthase A (P5CSA), which was upregulated and downregulated in comparison with wild-type plants, respectively. This enzyme is involved in the biosynthesis of proline an osmoprotective compound crucial for stress tolerance and plant growth and development [54]. Nevertheless, the analysis of proline content did not reveal any significant differences between the tested plant lines (Supplementary Fig S6). This aspect requires further investigation including enzymatic activity assays.

## Conclusions

The results presented in this study demonstrated that overexpression of one gene – *AtPDAT1* involved in triacylglycerol biosynthesis significantly changes proteome of Arabidopsis plants. Surprisingly, the increased level of *AtPDAT1* results in a much more pronounced phenotype than its complete deletion. Overexpression strongly affects the level of proteins dependent and independent on lipid metabolism. Observed changes in processes associated with membrane lipids metabolism, caused by *AtPDAT1* overexpression, indicated that its elevated turnover might be linked to processes such as photoprotection, autophagy and stress response. These processes provide plants with boosted vitality, fitness and indicate that plants are less susceptible to abiotic stress stimuli via improvement of described groups of proteins which we can call “lipid-associated proteins involved in stress”. Described proteins and mechanisms seem to be sufficient as major components for adaptation and potential abiotic stress response to keep plants in better condition than control plant. Other processes involved in adaptation and stress responses seems to be less prominent, mainly the level of the proteins involved in plant response to biotic stress are downregulated. Simultaneously, proteomic analysis of *pdat1* line did not reveal such broad protein changes caused by the knock-out mutation, as observed in the case of overexpression. In contrast to the overexpressor lines, *pdat1* line was characterized by increased accumulation of proteins involved in response to biotic stresses, and reduction in those involved in response to abiotic stress. In this study, we have also provided, in detail, simple and reproducible methodology for analysis of the plant leaf proteome to enhance the effectiveness of plant sample preparations for proteomics study.

## Supplementary Information

Supplementary Material 1.

Supplementary Material 2.

Supplementary Material 3.

## Data Availability

The datasets generated and analyzed during the current study are available; the mass spectrometry (MS) proteomics data have been deposited to the ProteomeXchange Consortium via the PRIDE partner repository with the dataset identifier PXD043240. Proline content analysis data set has been deposited: dx.doi.org/10.71853/d612-t891.
