# Supplementary material for: Quantitative proteomic analysis of Arabidopsis thaliana with different levels of phospholipid:diacylglycerol acyltransferase1 expression
Source: BMC Genomics. 2025 Sep 29;26:846. doi: 10.1186/s12864-025-12041-7 (PMC12482026; doi:10.1186/s12864-025-12041-7)
Supplement: Supplementary file 1 — Supplementary Material 1. [file 12864_2025_12041_MOESM1_ESM.pdf]

Quantitative proteomic analysis of *Arabidopsis thaliana* with different levels of p*hospholipid:diacylglycerol acyltransferase1* expression

Artur Piróg[1†], Sylwia Klińska-Bąchor[2*†], Bartosz Głąb[2], Sara Kędzierska[2], Katarzyna Jasieniecka-Gazarkiewicz[2], Antoni Banaś[2], Sachin Kote[*1]

[1] International Centre for Cancer Vaccine Science, University of Gdansk, 80-822, Gdańsk, Poland

[2] Intercollegiate Faculty of Biotechnology, University of Gdańsk and Medical University of Gdańsk, 80-307 Gdańsk, PL

*Correspondence author: sylwia.klinska@ug.edu.pl (Sylwia Klińska-Bąchor) and sachin.kote@ug.edu.pl (Sachin Kote)

A.P. and S.K-B. contributed equally to this work, and they are marked by †sign.

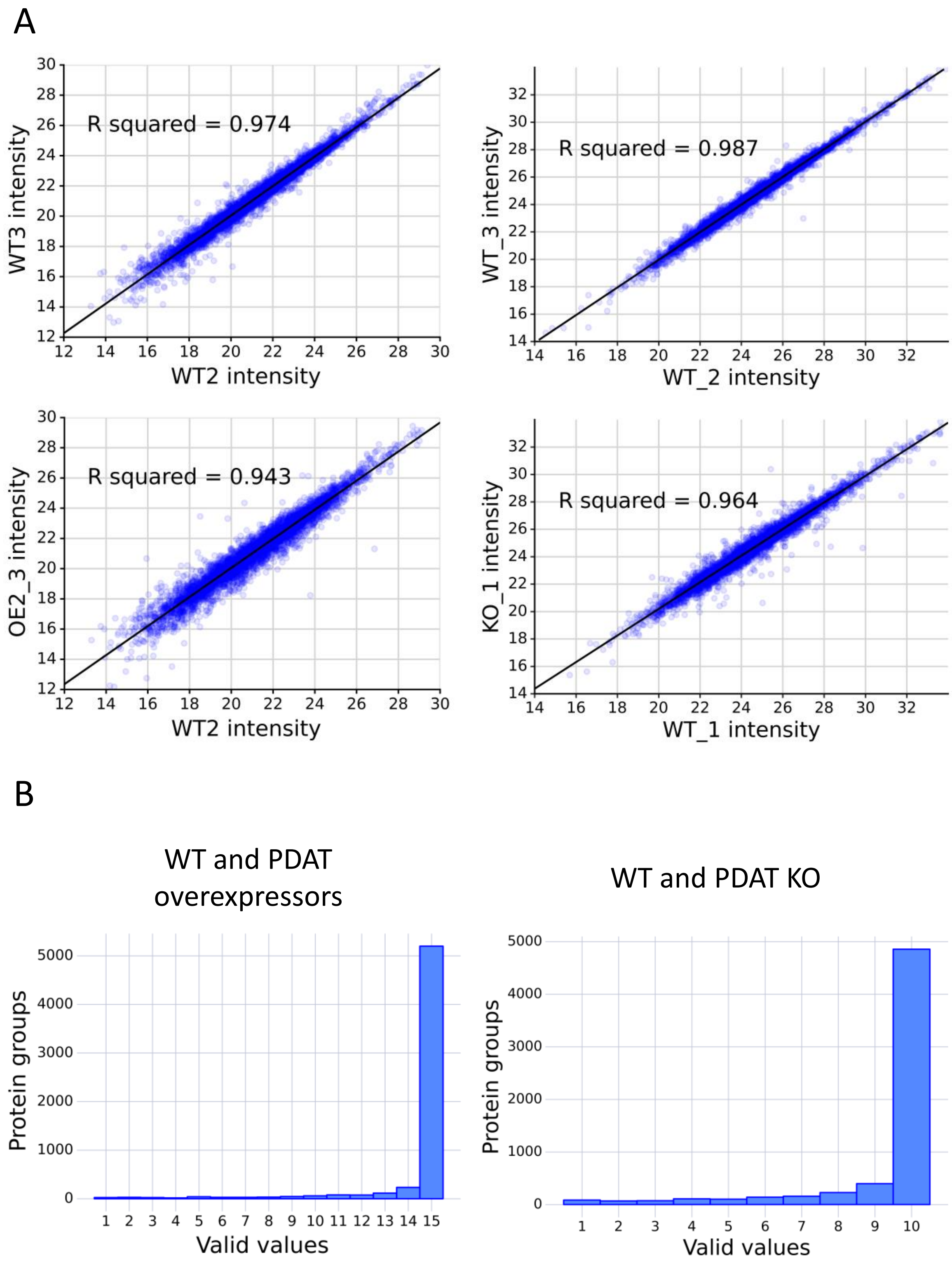

Figure S1. Overview of data quality. A) Reproducibility of protein quantification between representative pairs of replicates. B) Distribution of valid quantification values across the obtained dataset. 15 (overexpressors) or 10 (knock-out) valid values mean complete quantification without missing value.

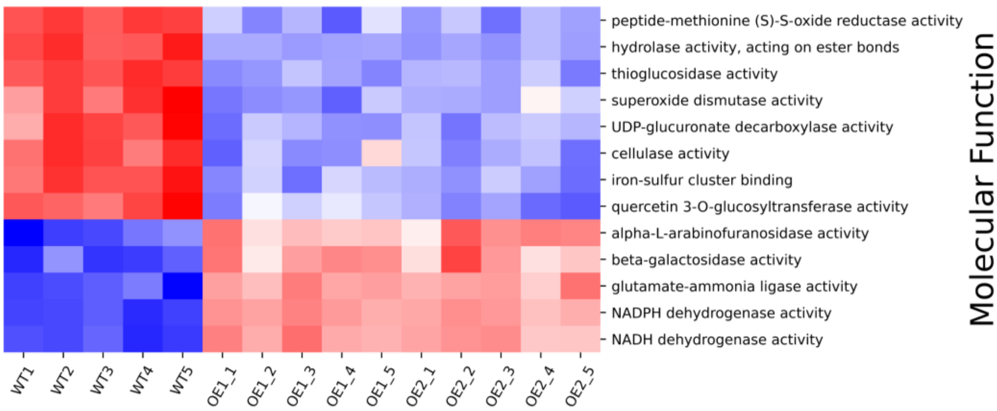

Weighted summarization

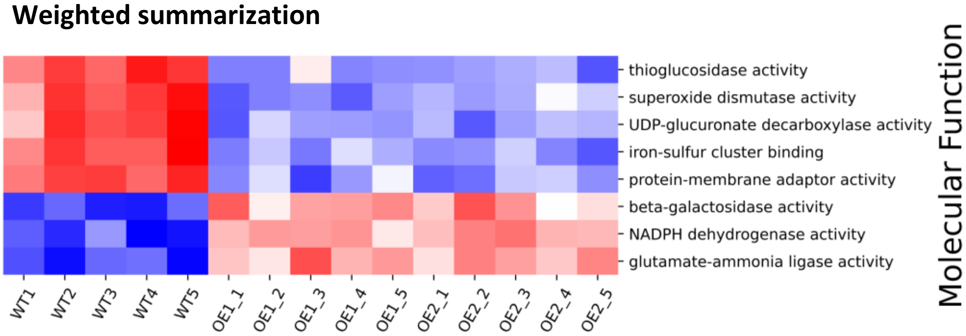

Non-weighted summarization

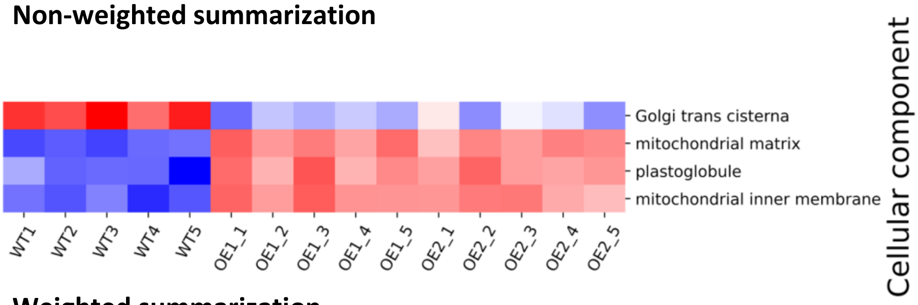

Weighted summarization

Non-weighted summarization - no relevant Cellular Component terms

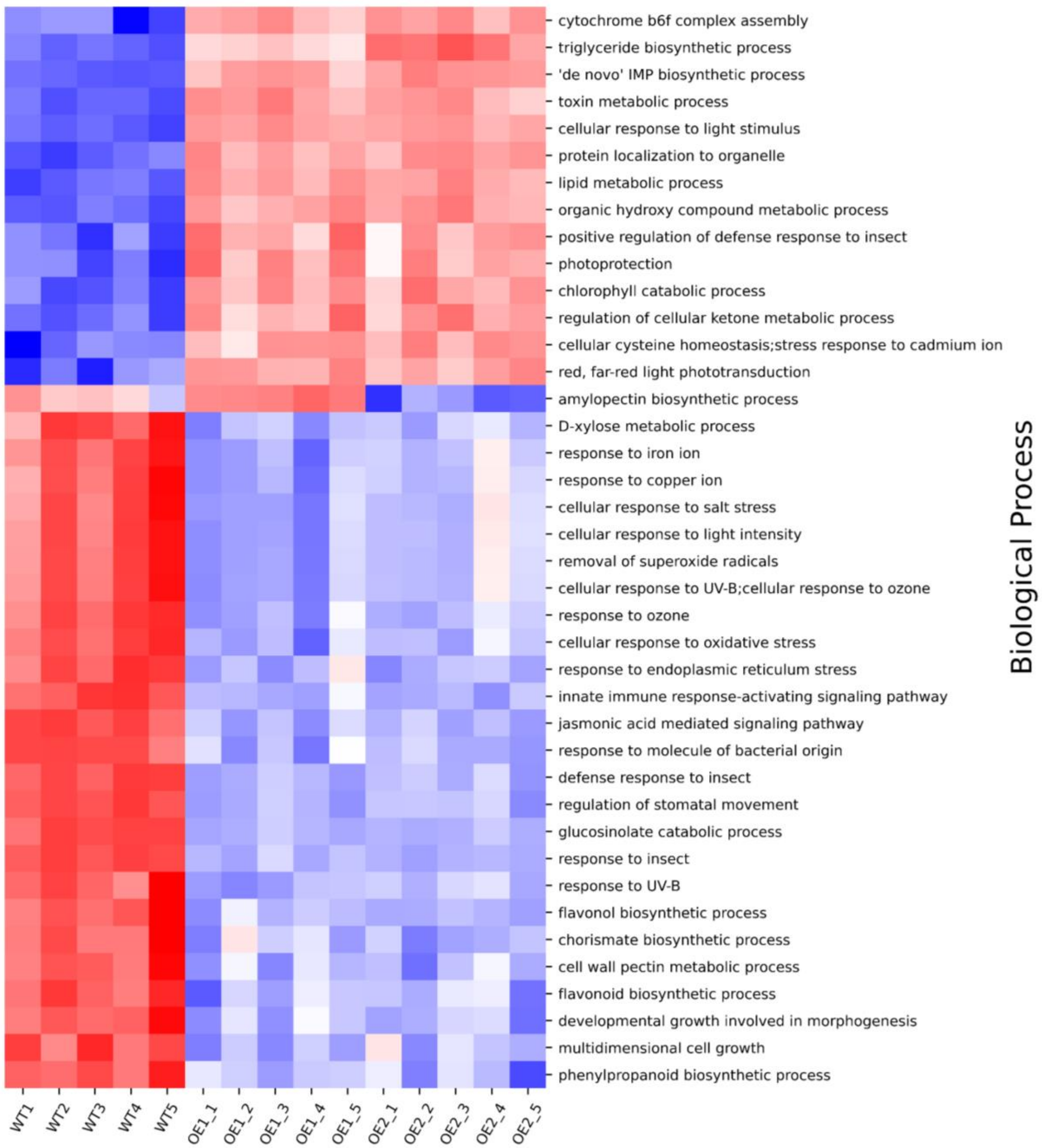

Weighted summarization

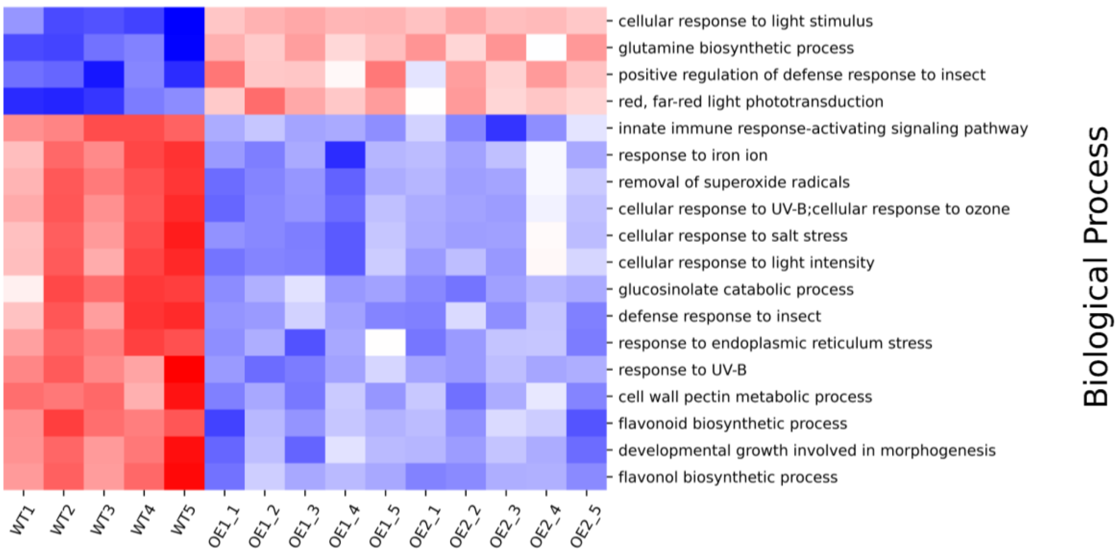

Non-weighted summarization

Figure S2. Overall visualization of affected Gene Ontology terms in *AtPDAT1* overexpressors. . Deatils for weighthing are described in Methods section. Red color indicates upregulation, blue color indicates downregulation. Filtering applied to visualization:

MF weighted : min. proteins - 4, max. ANOVA p-val : 10^{-5}, min. difference : 0.7
MF non-weighted : min. proteins - 4, max. ANOVA p-val : 10^{-5}, min. difference : 0.4
CC weighted : min. proteins - 3, max. ANOVA p-val : 10^{-5}, min. difference : 0.7
CC non-weighted : min. proteins - 3, max. ANOVA p-val : 10^{-5}, min. difference : 0.4
BP weighted : min. proteins - 4, max. ANOVA p-val : 10^{-5}, min. difference : 0.7
BP non-weighted : min. proteins - 4, max. ANOVA p-val : 10^{-5}, min. difference : 0.4

min.proteins - minimal number of proteins detected for particular term
max. ANOVA p-val - maximal uncorrected ANOVA p-value
min.difference - minimal difference of means between groups.

Biological Process

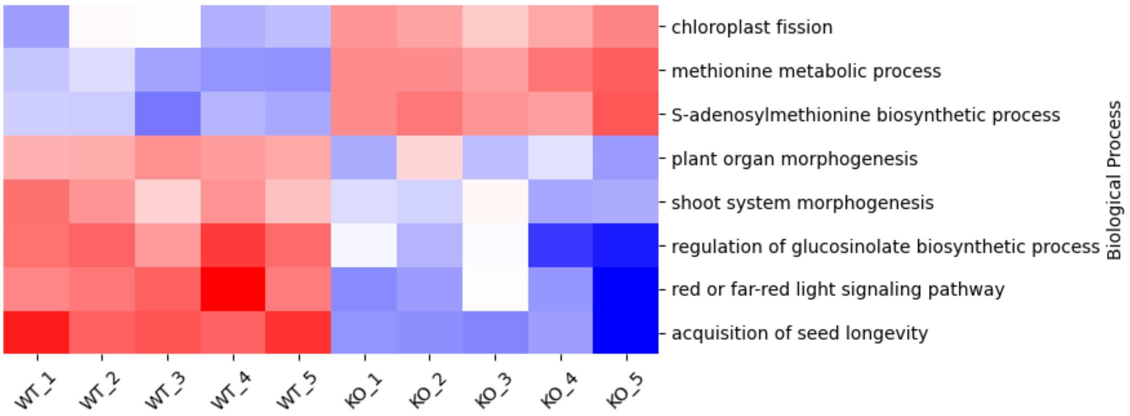

Molecular function

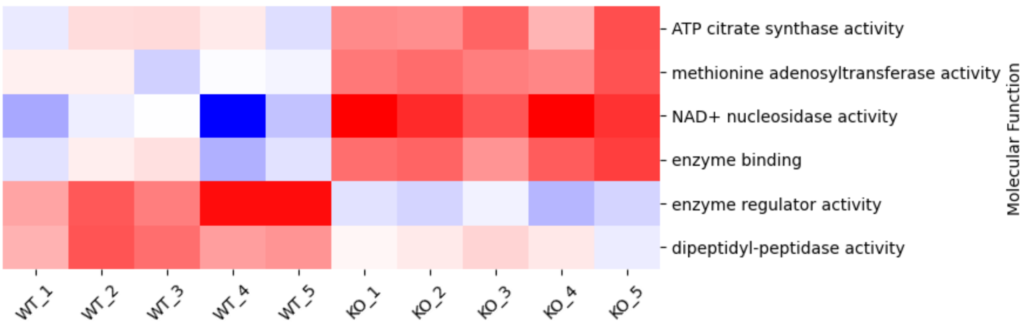

Cellular component

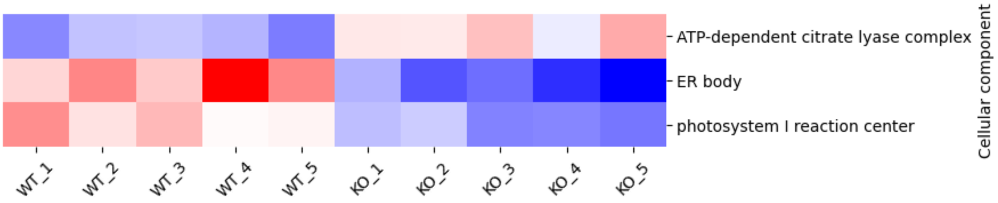

Figure S3 Overall visualization of affected Gene Ontology terms in *pdat1* (knock-out) plants. Red color indicates upregulation, blue color indicates downregulation. Filtering applied to visualization:

BP non-weighted : min. proteins - 4, max. ANOVA p-val : 10-3, min. difference : 0.25
MF non-weighted : min. proteins - 4, max. ANOVA p-val : 10-3, min. difference : 0.25
CC non-weighted : min. proteins - 4, max. ANOVA p-val : 10-3, min. difference : 0.25

Filtering was relaxed compared to *AtPDAT1* overexpression analysis.
min.proteins - minimal number of proteins detected for particular term
max. ANOVA p-val - maximal uncorrected ANOVA p-value
min.difference - minimal difference of means between groups.

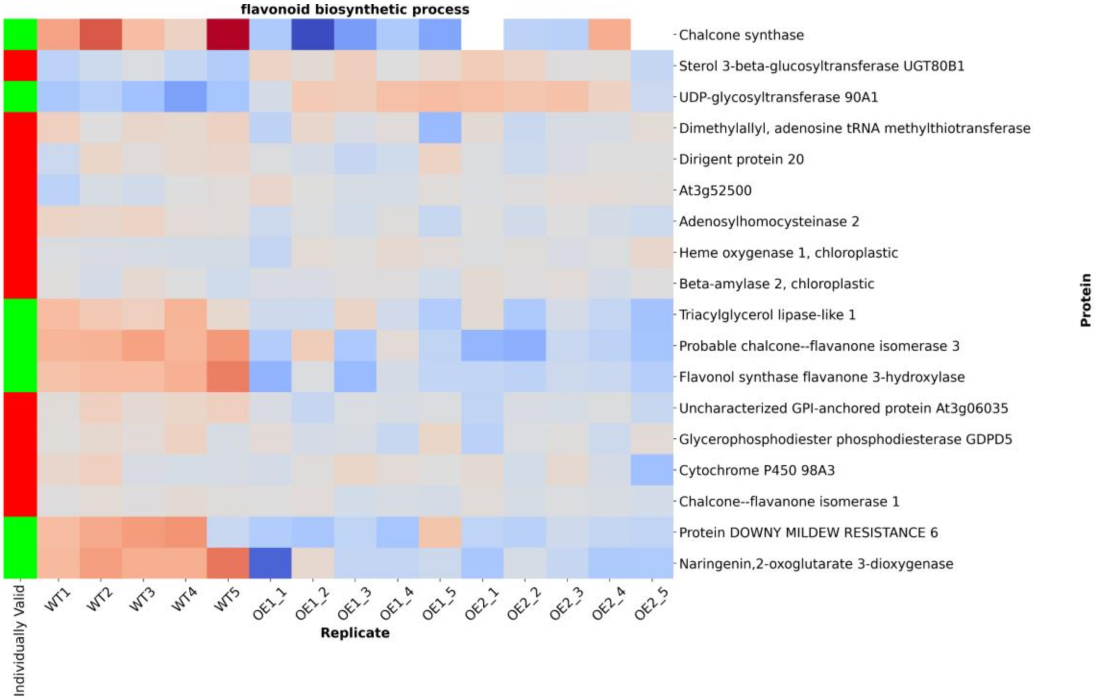
flavonoid biosynthetic process
Chalcone synthase
Sterol 3-beta-glucosyltransferase UGT80B1
UDP-glycosyltransferase 90A1
Dimethylallyl, adenosine tRNA methylthiotransferase
Dirigent protein 20
At3g52500
Adenosylhomocysteinase 2
Heme oxygenase 1, chloroplastic
Beta-amylase 2, chloroplastic
Triacylglycerol lipase-like 1
Probable chalcone--flavanone isomerase 3
Flavonol synthase flavanone 3-hydroxylase
Uncharacterized GPI-anchored protein At3g06035
Glycerophosphodiester phosphodiesterase GDPD5
Cytochrome P450 98A3
Chalcone--flavanone isomerase 1
Protein DOWNY MILDEW RESISTANCE 6
Naringenin,2-oxoglutarate 3-dioxygenase
Protein
Individually Valid
WT1
WT2
WT3
WT4
WT5
OE1_1
OE1_2
OE1_3
OE1_4
OE1_5
OE2_1
OE2_2
OE2_3
OE2_4
OE2_5
Replicate

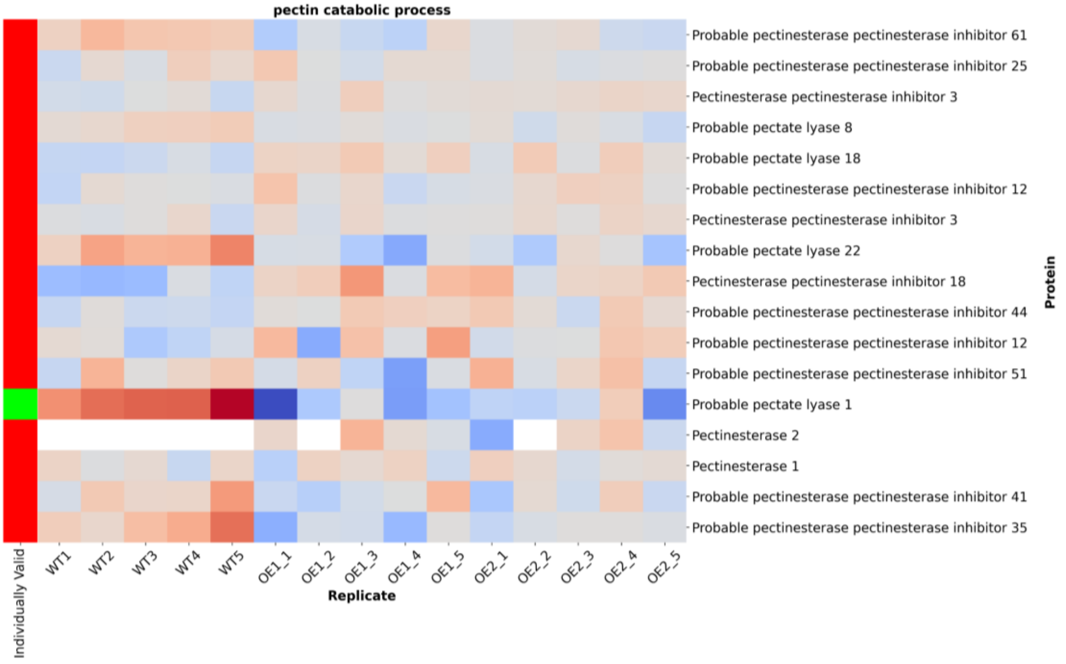
pectin catabolic process
Probable pectinesterase pectinesterase inhibitor 61
Probable pectinesterase pectinesterase inhibitor 25
Pectinesterase pectinesterase inhibitor 3
Probable pectate lyase 8
Probable pectate lyase 18
Probable pectinesterase pectinesterase inhibitor 12
Pectinesterase pectinesterase inhibitor 3
Probable pectate lyase 22
Pectinesterase pectinesterase inhibitor 18
Probable pectinesterase pectinesterase inhibitor 44
Probable pectinesterase pectinesterase inhibitor 12
Probable pectinesterase pectinesterase inhibitor 51
Probable pectate lyase 1
Pectinesterase 2
Pectinesterase 1
Probable pectinesterase pectinesterase inhibitor 41
Probable pectinesterase pectinesterase inhibitor 35
Protein
Individually Valid
WT1
WT2
WT3
WT4
WT5
OE1_1
OE1_2
OE1_3
OE1_4
OE1_5
OE2_1
OE2_2
OE2_3
OE2_4
OE2_5
Replicate

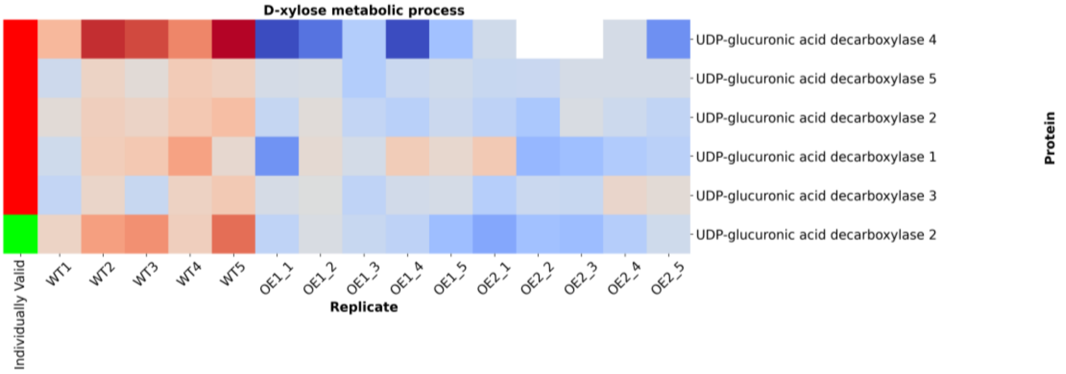
D-xylose metabolic process
UDP-glucuronic acid decarboxylase 4
UDP-glucuronic acid decarboxylase 5
UDP-glucuronic acid decarboxylase 2
UDP-glucuronic acid decarboxylase 1
UDP-glucuronic acid decarboxylase 3
UDP-glucuronic acid decarboxylase 2
Protein
Individually Valid
WT1
WT2
WT3
WT4
WT5
OE1_1
OE1_2
OE1_3
OE1_4
OE1_5
OE2_1
OE2_2
OE2_3
OE2_4
OE2_5
Replicate

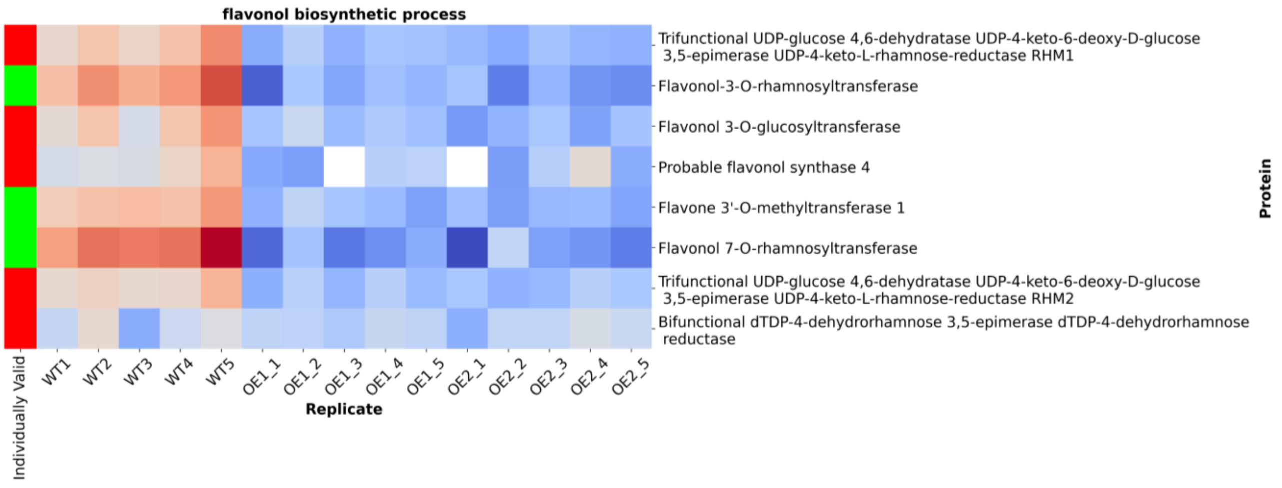
flavonol biosynthetic process
Trifunctional UDP-glucose 4,6-dehydratase UDP-4-keto-6-deoxy-D-glucose 3,5-epimerase UDP-4-keto-L-rhamnose-reductase RHM1
Flavonol-3-O-rhamnosyltransferase
Flavonol 3-O-glucosyltransferase
Probable flavonol synthase 4
Flavone 3'-O-methyltransferase 1
Flavonol 7-O-rhamnosyltransferase
Trifunctional UDP-glucose 4,6-dehydratase UDP-4-keto-6-deoxy-D-glucose 3,5-epimerase UDP-4-keto-L-rhamnose-reductase RHM2
Bifunctional dTDP-4-dehydrorhamnose 3,5-epimerase dTDP-4-dehydrorhamnose reductase
Protein
Individually Valid
WT1
WT2
WT3
WT4
WT5
OE1_1
OE1_2
OE1_3
OE1_4
OE1_5
OE2_1
OE2_2
OE2_3
OE2_4
OE2_5
Replicate

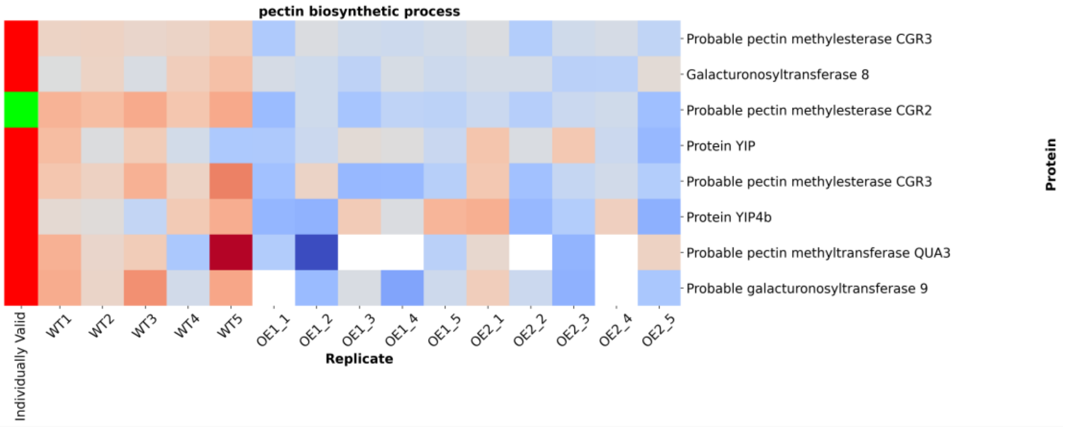
pectin biosynthetic process
Probable pectin methylesterase CGR3
Galacturonosyltransferase 8
Probable pectin methylesterase CGR2
Protein YIP
Probable pectin methylesterase CGR3
Protein YIP4b
Probable pectin methyltransferase QUA3
Probable galacturonosyltransferase 9
Protein
Individually Valid
WT1
WT2
WT3
WT4
WT5
OE1_1
OE1_2
OE1_3
OE1_4
OE1_5
OE2_1
OE2_2
OE2_3
OE2_4
OE2_5
Replicate

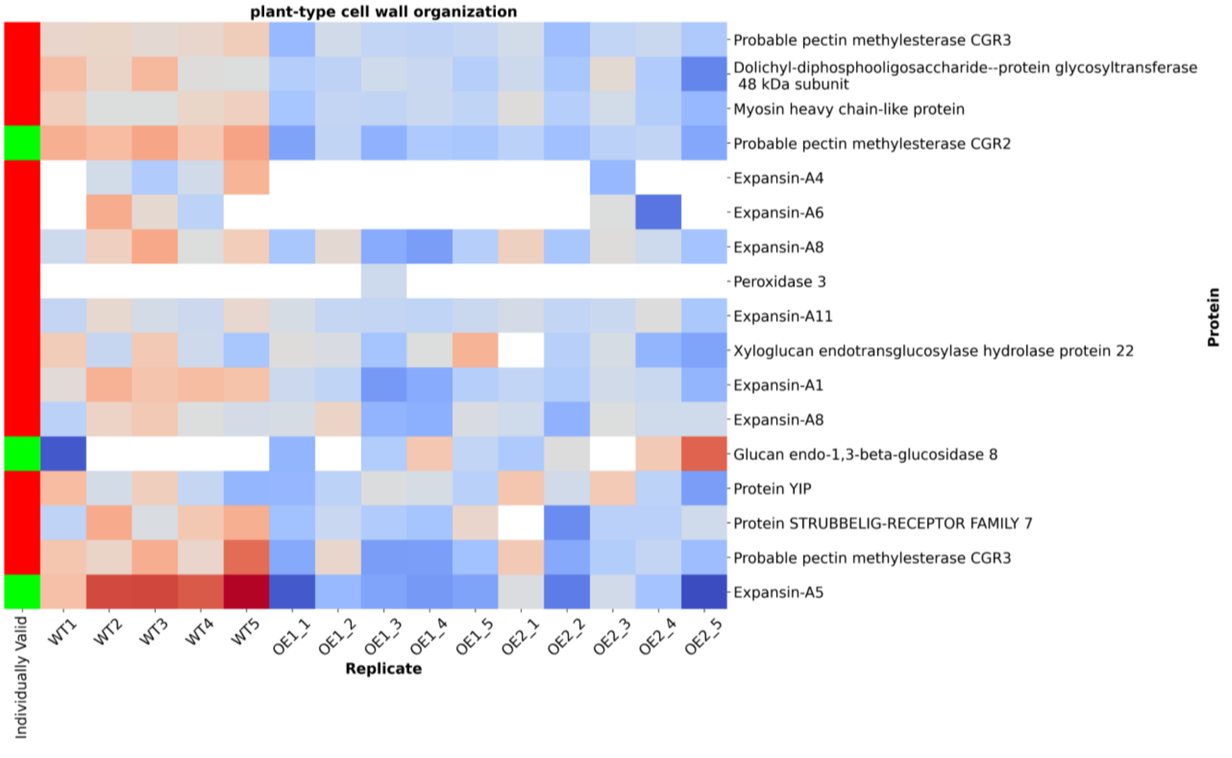
plant-type cell wall organization
Probable pectin methylesterase CGR3
Dolichyl-diphosphooligosaccharide--protein glycosyltransferase 48 kDa subunit
Myosin heavy chain-like protein
Probable pectin methylesterase CGR2
Expansin-A4
Expansin-A6
Expansin-A8
Peroxidase 3
Expansin-A11
Xyloglucan endotransglucosylase hydrolase protein 22
Expansin-A1
Expansin-A8
Glucan endo-1,3-beta-glucosidase 8
Protein YIP
Protein STRUBBELIG-RECEPTOR FAMILY 7
Probable pectin methylesterase CGR3
Expansin-A5
Protein
Individually Valid
WT1
WT2
WT3
WT4
WT5
OE1_1
OE1_2
OE1_3
OE1_4
OE1_5
OE2_1
OE2_2
OE2_3
OE2_4
OE2_5
Replicate

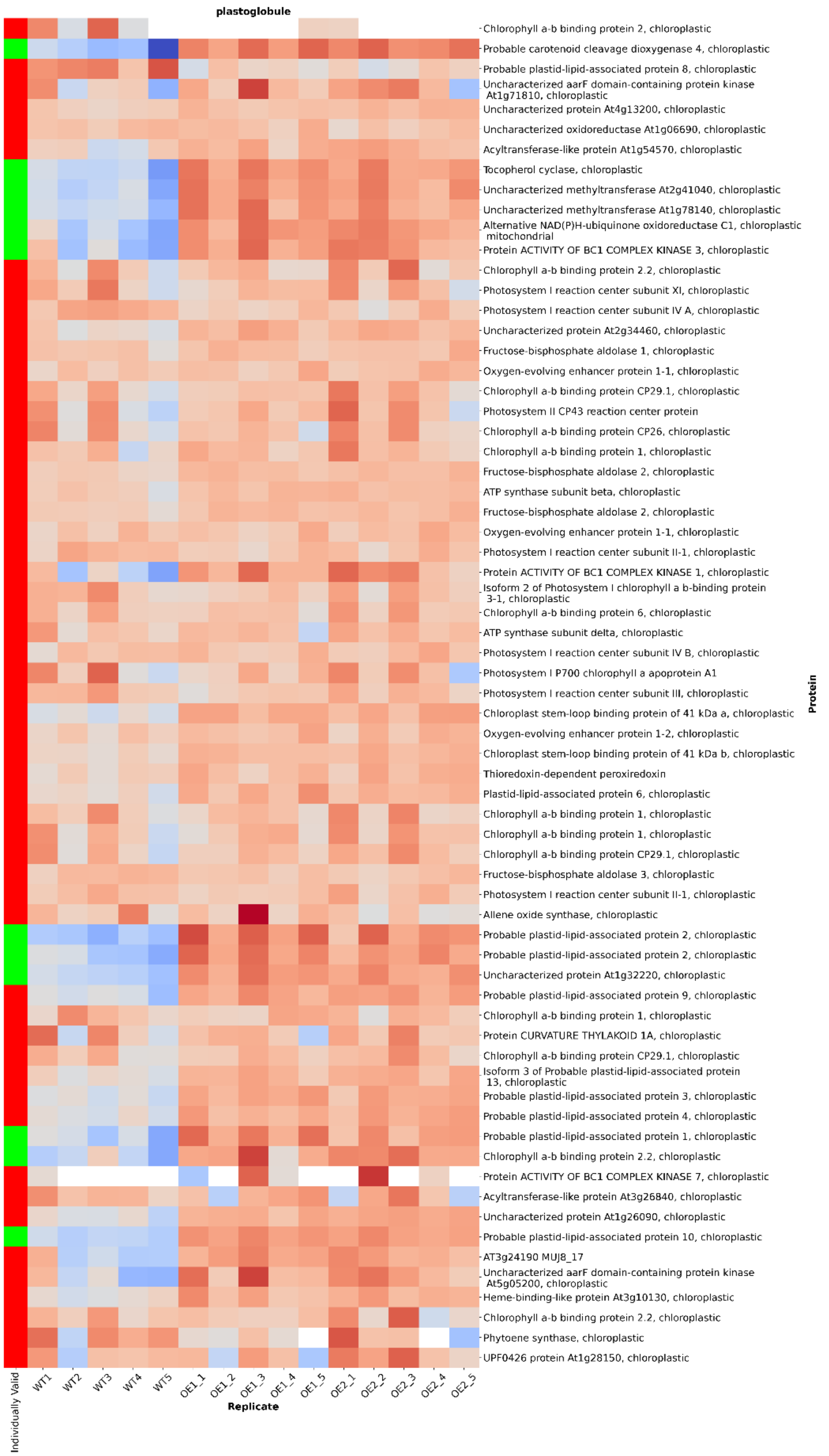
plastoglobule
Chlorophyll a-b binding protein 2, chloroplastic
Probable carotenoid cleavage dioxygenase 4, chloroplastic
Probable plastid-lipid-associated protein 8, chloroplastic
Uncharacterized aarF domain-containing protein kinase At1g71810, chloroplastic
Uncharacterized protein At4g13200, chloroplastic
Uncharacterized oxidoreductase At1g06690, chloroplastic
Acyltransferase-like protein At1g54570, chloroplastic
Tocopherol cyclase, chloroplastic
Uncharacterized methyltransferase At2g41040, chloroplastic
Uncharacterized methyltransferase At1g78140, chloroplastic
Alternative NAD(P)H-ubiquinone oxidoreductase C1, chloroplastic mitochondrial
Protein ACTIVITY OF BC1 COMPLEX KINASE 3, chloroplastic
Chlorophyll a-b binding protein 2.2, chloroplastic
Photosystem I reaction center subunit XI, chloroplastic
Photosystem I reaction center subunit IV A, chloroplastic
Uncharacterized protein At2g34460, chloroplastic
Fructose-bisphosphate aldolase 1, chloroplastic
Oxygen-evolving enhancer protein 1-1, chloroplastic
Chlorophyll a-b binding protein CP29.1, chloroplastic
Photosystem II CP43 reaction center protein
Chlorophyll a-b binding protein CP26, chloroplastic
Chlorophyll a-b binding protein 1, chloroplastic
Fructose-bisphosphate aldolase 2, chloroplastic
ATP synthase subunit beta, chloroplastic
Fructose-bisphosphate aldolase 2, chloroplastic
Oxygen-evolving enhancer protein 1-1, chloroplastic
Photosystem I reaction center subunit II-1, chloroplastic
Protein ACTIVITY OF BC1 COMPLEX KINASE 1, chloroplastic
Isoform 2 of Photosystem I chlorophyll a b-binding protein 3-1, chloroplastic
Chlorophyll a-b binding protein 6, chloroplastic
ATP synthase subunit delta, chloroplastic
Photosystem I reaction center subunit IV B, chloroplastic
Photosystem I P700 chlorophyll a apoprotein A1
Photosystem I reaction center subunit III, chloroplastic
Chloroplast stem-loop binding protein of 41 kDa a, chloroplastic
Oxygen-evolving enhancer protein 1-2, chloroplastic
Chloroplast stem-loop binding protein of 41 kDa b, chloroplastic
Thioredoxin-dependent peroxiredoxin
Plastid-lipid-associated protein 6, chloroplastic
Chlorophyll a-b binding protein 1, chloroplastic
Chlorophyll a-b binding protein 1, chloroplastic
Chlorophyll a-b binding protein CP29.1, chloroplastic
Fructose-bisphosphate aldolase 3, chloroplastic
Photosystem I reaction center subunit II-1, chloroplastic
Allene oxide synthase, chloroplastic
Probable plastid-lipid-associated protein 2, chloroplastic
Probable plastid-lipid-associated protein 2, chloroplastic
Uncharacterized protein At1g32220, chloroplastic
Probable plastid-lipid-associated protein 9, chloroplastic
Chlorophyll a-b binding protein 1, chloroplastic
Protein CURVATURE THYLAKOID 1A, chloroplastic
Chlorophyll a-b binding protein CP29.1, chloroplastic
Isoform 3 of Probable plastid-lipid-associated protein 13, chloroplastic
Probable plastid-lipid-associated protein 3, chloroplastic
Probable plastid-lipid-associated protein 4, chloroplastic
Probable plastid-lipid-associated protein 1, chloroplastic
Chlorophyll a-b binding protein 2.2, chloroplastic
Protein ACTIVITY OF BC1 COMPLEX KINASE 7, chloroplastic
Acyltransferase-like protein At3g26840, chloroplastic
Uncharacterized protein At1g26090, chloroplastic
Probable plastid-lipid-associated protein 10, chloroplastic
AT3g24190 MUJ8_17
Uncharacterized aarF domain-containing protein kinase At5g05200, chloroplastic
Heme-binding-like protein At3g10130, chloroplastic
Chlorophyll a-b binding protein 2.2, chloroplastic
Phytoene synthase, chloroplastic
UPF0426 protein At1g28150, chloroplastic
Protein
Individually Valid
WT1
WT2
WT3
WT4
WT5
OE1_1
OE1_2
OE1_3
OE1_4
OE1_5
OE2_1
OE2_2
OE2_3
OE2_4
OE2_5
Replicate

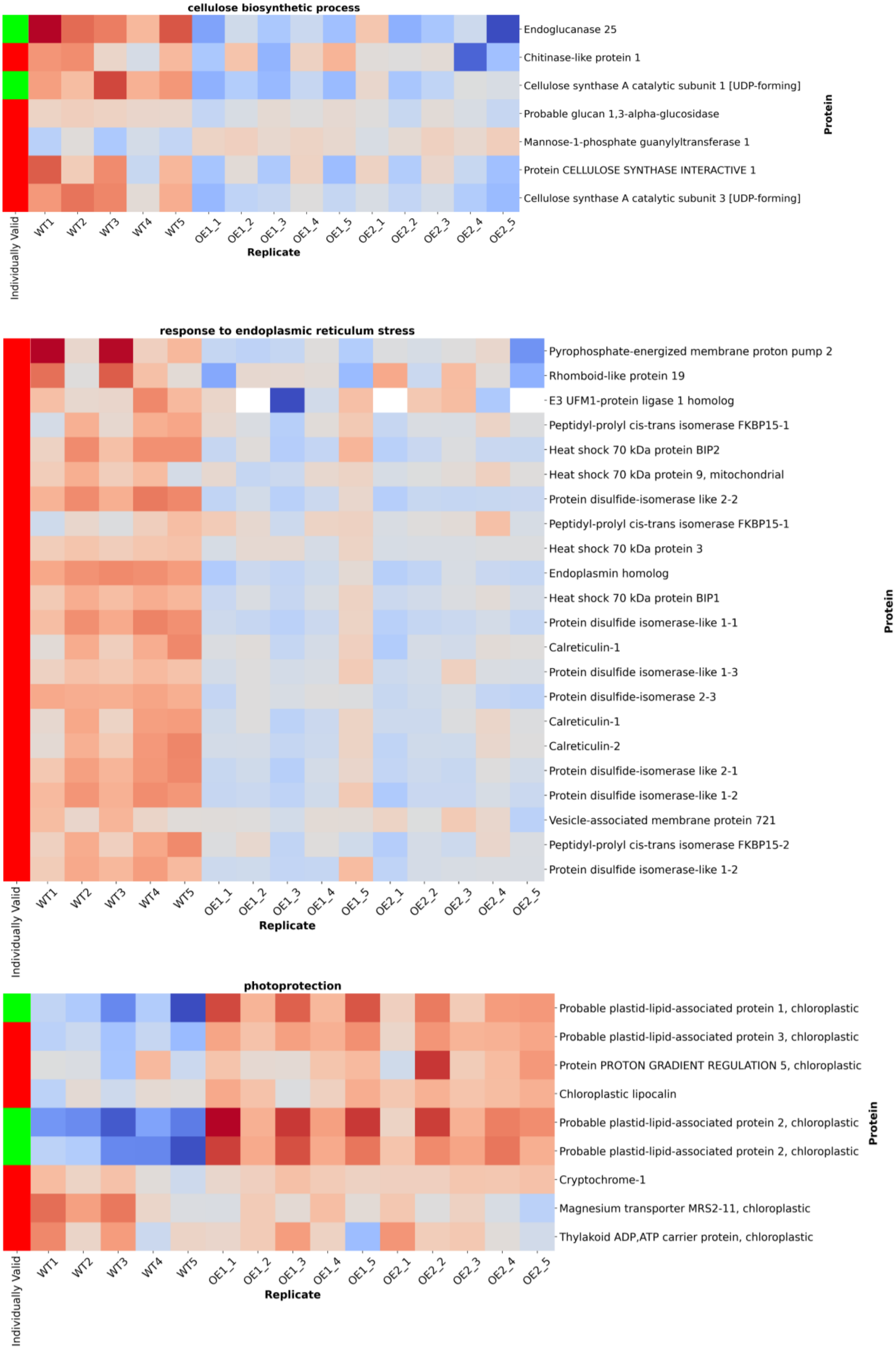

Figure S4. Selected Gene Ontology terms visualized on protein level. Blue color indicates lower expression and orange color indicates higher expression. White color indicates presence of missing value after MaxLFQ quantification. Green mark in first column indicates that protein group was accepted as significantly regulated individually.

A.) *At*PDAT1 overexpression

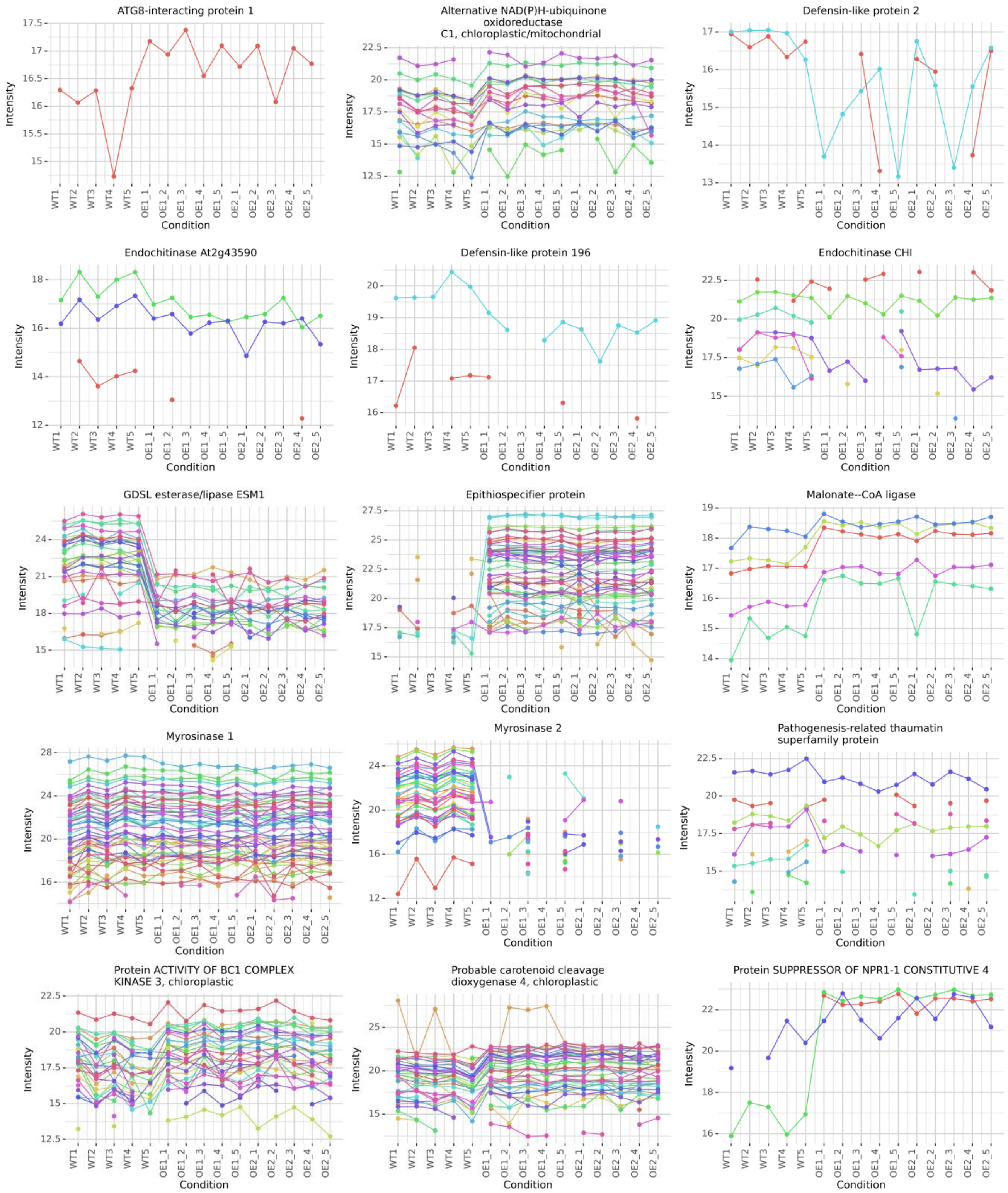

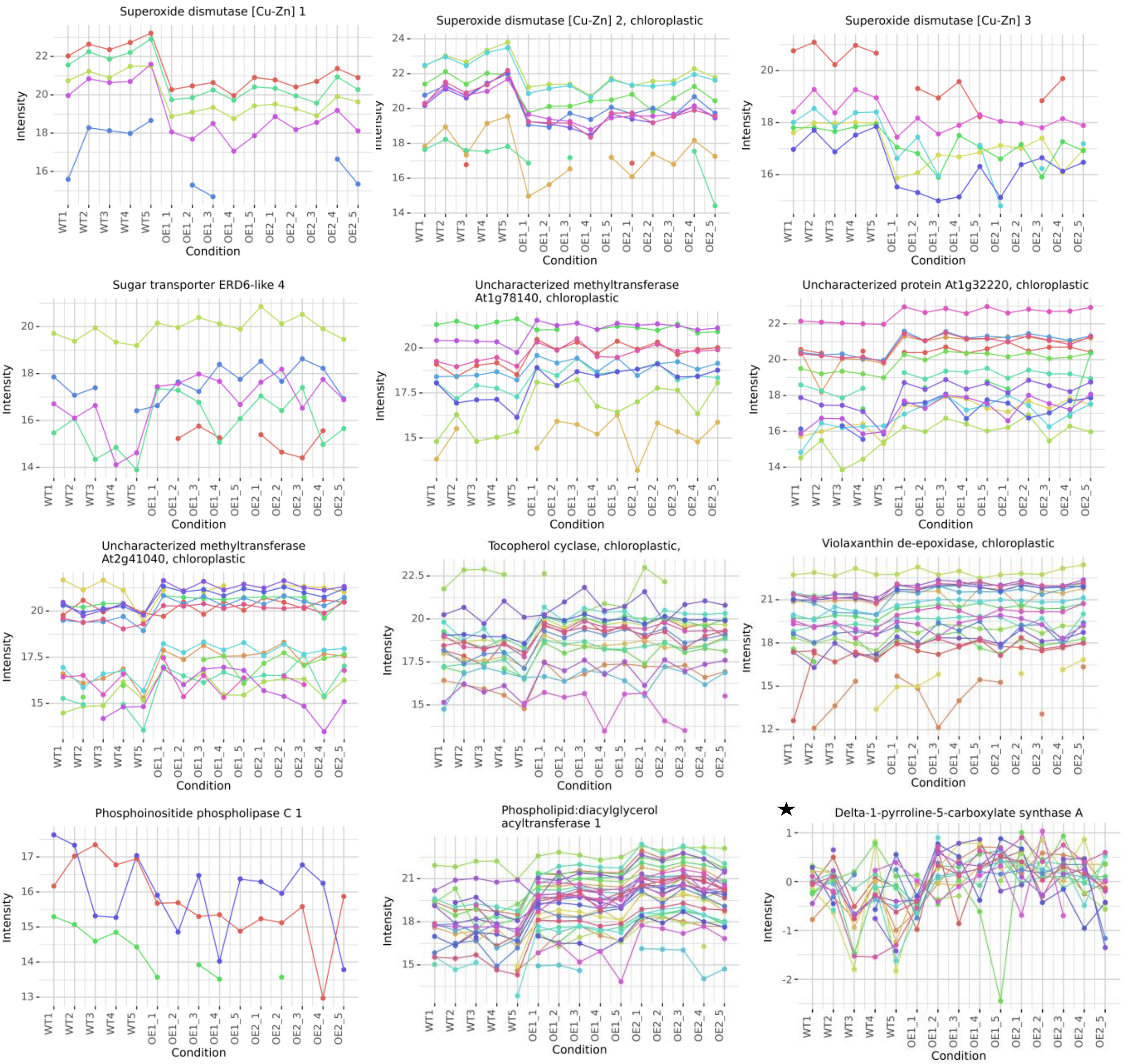

★ Values were mean-subtracted for enhanced visualization of differences

B.) *pdat1* knockout

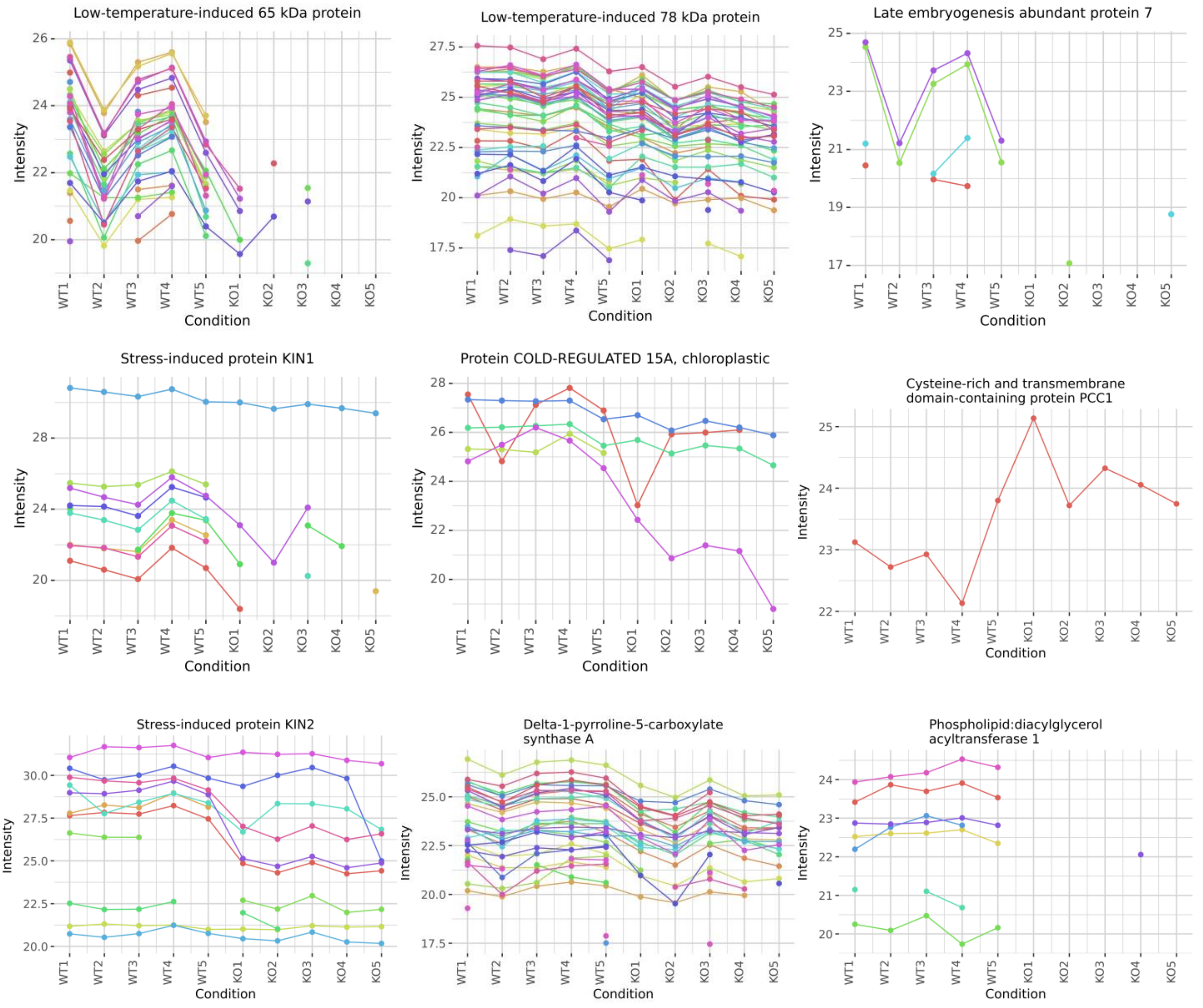

Figure S5. Intensity plots of peptides belonging to proteins mentioned in the main text.

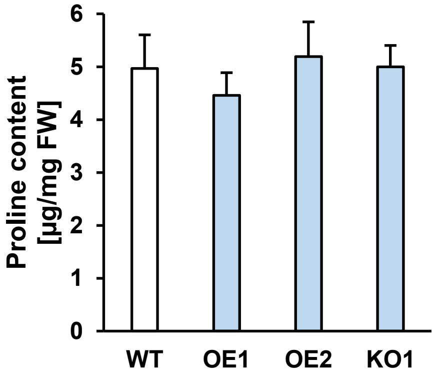

Figure S6. Proline content in the tested *A. thaliana PDAT1* overexpressing (OE) and knock-out (KO) lines. (WT – wild-type; FW – fresh weight).
